# HSF1-dependent and -independent regulation of the mammalian *in vivo* heat shock response and its impairment in Huntington's disease mouse models

**DOI:** 10.1038/s41598-017-12897-0

**Published:** 2017-10-02

**Authors:** Andreas Neueder, Theresa A. Gipson, Sophie Batterton, Hayley J. Lazell, Pamela P. Farshim, Paolo Paganetti, David E. Housman, Gillian P. Bates

**Affiliations:** 1 UCL Huntington’s Disease Centre, Sobell Department of Motor Neuroscience, UCL Institute of Neurology, University College London, London, United Kingdom; 2 Koch Institute for Integrative Cancer Research, Massachusetts Institute of Technology, Cambridge, MA 02139 United States; 3 Neuroscience Discovery, Novartis Institutes for Biomedical Research, CH-4002 Basel, Switzerland; 4 Present Address: Laboratory for Biomedical Neuroscience, Neurocenter of Southern Switzerland, EOC, c/o SIRM, Torricella-Taverne, Switzerland

## Abstract

The heat shock response (HSR) is a mechanism to cope with proteotoxic stress by inducing the expression of molecular chaperones and other heat shock response genes. The HSR is evolutionarily well conserved and has been widely studied in bacteria, cell lines and lower eukaryotic model organisms. However, mechanistic insights into the HSR in higher eukaryotes, in particular in mammals, are limited. We have developed an *in vivo* heat shock protocol to analyze the HSR in mice and dissected heat shock factor 1 (HSF1)-dependent and -independent pathways. Whilst the induction of proteostasis-related genes was dependent on HSF1, the regulation of circadian function related genes, indicating that the circadian clock oscillators have been reset, was independent of its presence. Furthermore, we demonstrate that the *in vivo* HSR is impaired in mouse models of Huntington’s disease but we were unable to corroborate the general repression of transcription that follows a heat shock in lower eukaryotes.

## Introduction

The heat shock response (HSR) is a defense program for cells to counter the proteotoxic effects of elevated temperatures and other stressors. Heat shock transcription factors (HSFs) are the main transcription factors responsible for the induction of heat shock response genes. In mammals, seven members of the HSF family have been identified, many of which exist in several isoforms, e.g. HSF1 has four isoforms^1^. HSF1 is not only required for induction of the HSR, but is also an important player in processes like ageing, energy metabolism and carcinogenesis^2^.

The transient induction of molecular chaperones (heat shock proteins, HSPs) is beneficial in many neurodegenerative diseases due to their protection against the accumulation of misfolded proteins^3^, but the constitutive activation of the HSR into a ‘maladaptive stress response’ negatively impacts the proteostasis networks of a diseased cell^4^. This observation seems counterintuitive, as several pathways, including the HSR, are impaired in protein folding diseases, e.g. Huntington’s disease (HD)^5,6^. Activation of the HSR through a small molecule in mouse models of HD was shown to have transiently beneficial effects on disease phenotypes, but the ability to induce the HSR became impaired with disease progression^7^. The molecular basis for this is potentially a change in the chromatin structure of heat shock protein promoters leading to reduced HSF1 binding and recruitment or release of RNA polymerase II at these genes^7^. Supporting this, the genome wide binding pattern of HSF1 is drastically changed in a cell model of HD, interestingly however only under heat shock conditions^8^.

Recent publications have shed more light on the roles of HSF1 in various cellular and systemic processes. During carcinogenesis, HSF1 regulates a non-heat shock response gene network^9^. The transcription factors E2F and HSF1 orchestrate expression of a network of genes required for organismal development that is different from the HSR^10^. Solis and colleagues showed that yeast Hsf1 is required under normal conditions to inhibit protein aggregation and is only required to induce chaperone genes under heat stress. The majority of other heat shock response genes were Hsf1-independent^11^. A similar phenomenon was observed by Mahat et al., who found that only a fraction of the heat shock induced genes are bound by HSF1, and that HSF1 is dispensable for the induction of a large percentage of heat shock response genes in immortalized mouse embryonic fibroblast cell lines. They also observed that cytoskeletal genes were rapidly induced by heat shock in an HSF1-independent manner. Bioinformatic analyses identified candidate transcription factors that might mediate this HSF1-independent response including SRF, E2F and ELF1^12^.

This research has almost exclusively been conducted in cell lines and lower eukaryotic organisms. While these models provide valuable insights into HSR regulation, the findings might not translate directly into the mechanisms that control the HSR in higher eukaryotes. One example for this discrepancy is the decline of the proteostasis network with age, which is clearly evident in *C. elegans* ^13^, but is not a general feature in mice^14^. To examine mechanistic differences in the regulation of the HSR in mammals, and to analyze HSF1-dependent and -independent effects, we developed an *in vivo* heat shock protocol to induce a robust HSR in mice. Skeletal muscle atrophy, HTT aggregation, transcriptional dysregulation, weakness and contractile abnormalities are well-characterized phenotypes in both the R6/2 transgenic and *Hdh*Q150 knock-in models of HD. We compared the genome wide transcriptional response to heat shock in the *quadriceps femoris* of wild type mice with that of HD mouse models, and demonstrated a disease-related impairment of the *in vivo* HSR. We showed that some features of the HSR in cell models and lower eukaryotes are not recapitulated in mammals e.g. a general repression of transcription in response to heat shock. We observed an HSF1-independent regulation of circadian function related genes following heat shock, indicative of a mechanism for resetting circadian clock oscillators. A comprehensive understanding of the HSR in mammals is a prerequisite for the development of targeted strategies to manipulate this process.

## Results

The main questions we wanted to answer in this study were: What is the transcriptional response induced by an *in vivo* heat shock in mammals? What is the representation of the HSR impairment in HD mouse models when induced via *in vivo* heat stress? Which genes are HSF1 dependently and independently regulated? Furthermore, we used HSP90 inhibition with a small molecule (HSP990) as an alternative way to induce chaperone gene expression and to study potential differences between this and a heat shock. For all comparisons we analyzed the transcriptomic changes, protein induction of HSPs, and used bioinformatics to predict chromatin states and upstream regulators.

### Robust induction of an *in vivo* heat shock response in mice

**Figure 1:**
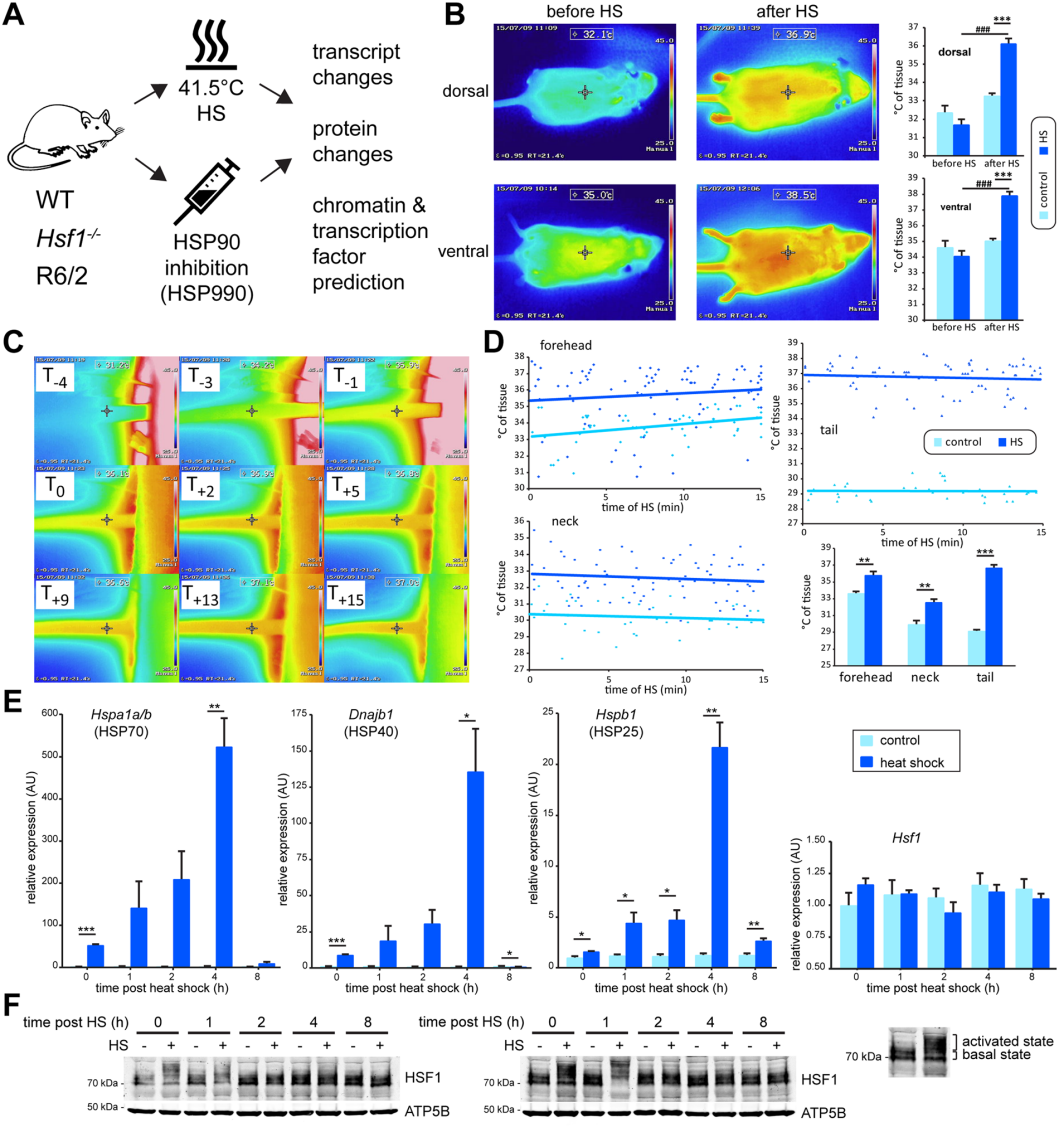
Development of an *in vivo* heat shock protocol. (**A**) Schematic depicting the treatment and processing pipeline used in this publication. WT = wild type; *Hsf1* ^−/−^ = *Hsf1* homozygous knockout mice; R6/2 = mouse model for Huntington’s disease. (**B**) Thermal camera images of exemplary mice before or after heat shock from the dorsal and ventral side. Control mice (control) were kept at 36.9 °C; heat shocked mice (HS) were subjected to a 15 minute heat shock at 41.5 °C. Quantification of the data is shown (right panel). Data are mean ± SEM; n ≥ 4; two-way ANOVA with Tukey *post hoc* test. (**C**) Time course of tail temperature (mins) from start of the procedure (T_-4_), beginning of the 15 minutes (T_0_) until the end of the heat shock (T_+15_). (**D**) Temperature differences as measured by thermal camera imaging between control and heat shocked animals. Quantification (lower right panel) was done by calculating the average temperature for each mouse across the treatment. Data are mean ± SEM; n ≥ 4 for forehead and neck, n ≥ 3 for tail; two-tailed homoscedastic Student’s *t*-test. (**E**) Kinetics of transcript induction of 3 HSP genes (*Hspa1a/b*, *Dnajb1*, *Hspb1*) and *Hsf1* from 0 to 8 hours after heat shock in *quadriceps femoris* muscle of 12 week old wild type mice. Data are mean ± SEM relative to the expression levels of each gene in the respective untreated group at 0 hours; n = 4; two-tailed homoscedastic Student’s *t*-test. (F) HSF1 protein analysis of the same samples as in (**E**). Both panel shows exemplary results for each time point. ATP5B was used as a loading control. Treatment: *p < 0.05, **p < 0.01, ***p < 0.001 and pre- / post-HS ^###^ *p* < 0.001.

We used a heat pad to quickly (~ 6 minutes) raise the core body temperature of mice to 41.5 °C ± 0.2 °C (Fig. 1A). The heat shock lasted for 15 minutes and was controlled by a rectal thermal probe. Control mice were kept at 36.9 °C ± 0.2 °C throughout the procedure. We found a significantly higher surface temperature, both dorsal and ventral, after heat shock in the treated mice (Fig. 1B). Thermal imaging clearly showed a constantly elevated temperature when imaging the tail (Fig. 1C). Quantification of thermal imaging data throughout the 15 minutes of treatment showed a clear distinction between controls and heat shock (HS) treated mice (Fig. 1D). To further characterize the kinetics of HSP induction, we analyzed the mRNA levels of 3 major HSP genes (*Hspa1a/b*, *Dnajb1*, *Hspb1*) and *Hsf1* immediately after the treatment, and up to 8 hours post treatment in *quadriceps femoris* muscles from wild type mice (Fig. 1E). All 3 HSP mRNAs showed increasing levels of expression up to 4 hours post treatment, which had rapidly declined by 8 hours. *Hsf1* mRNA levels remained unchanged. HSF1 undergoes major posttranslational modification upon heat stress, which can be visualized by a shift in apparent mass by western blotting. As expected, the activation (mass shift) of HSF1 preceded the major peak in HSR gene induction (see shift at 0 h and 1 h in Fig. 1F). HSF1 had already reverted to its basal state by 2 hours post heat shock (Fig. 1F). To gain an initial impression of the dependence of HSP gene expression on HSF1, we analyzed the expression levels of a more diverse set of HSP genes 4 hours post heat shock (Figure S1A) or HSP90 inhibition (Figure S1B) in wild type and *Hsf1* knockout mice. While all HSP genes, with the exception of *Hsp90aa1*, were induced by both stresses in wild type mice, we did not observe induction of HSP mRNAs in the *Hsf1* knockout mice. In summary, our *in vivo* heat shock procedure robustly induced a HSR in mice and we showed that HSP gene induction was dependent on mammalian HSF1.

### The HSR is impaired in mouse models of HD

**Figure 2:**
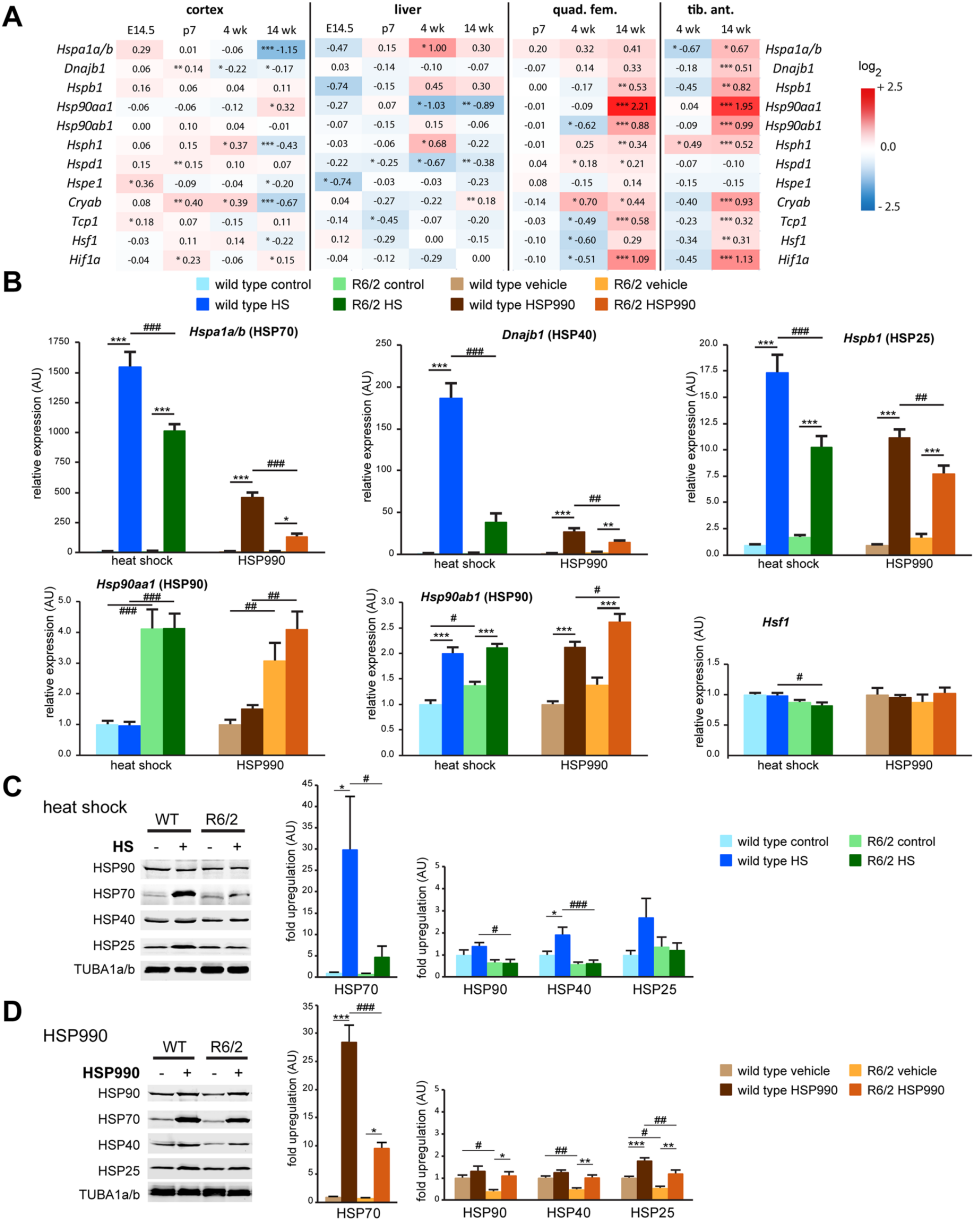
The heat shock response is impaired in the R6/2 mouse model of Huntington’s disease. (**A**) Heat map showing the basal transcript levels of several heat shock response genes and regulators in cortex, liver, *quadriceps femoris* (quad. fem.) and *tibialis anterior* (tib. ant.) muscles at different stages (E14.5 = embryonic day 14.5; p7 = postnatal day 7; wk = weeks). Data are the log_2_ fold changes of R6/2 vs. wild type mice. Data are mean ± SEM; n = 4; two-tailed homoscedastic Student’s *t*-test. Genotype: *p < 0.05, **p < 0.01, ***p < 0.001. (**B**) Transcript induction at 4 hours after heat shock or HSP90 inhibition (HSP990) in *quadriceps femoris* muscle of R6/2 and wild type mice at 12 week of age. Data are mean ± SEM relative to the levels of control or vehicle treated wild type animals; n ≥ 6; two-way ANOVA with Tukey *post hoc* test. (**C** and **D**) Heat shock protein induction at 24 hours after heat shock (HS) (**C**) or HSP90 inhibition (HSP990) (**D**) in *quadriceps femoris* muscle of R6/2 and wild type mice at 12 week of age. Data are mean ± SEM relative to the levels of control or vehicle treated wild type animals; n ≥ 6; two-way ANOVA with Tukey *post hoc* test. TUBA1a/b was used as a loading control. (**B**,**C** and **D**) Treatment: *p < 0.05, **p < 0.01, ***p < 0.001 and treatment/genotype ^#^ *p* < 0.05, ^##^ *p* < 0.01, ^###^ *p* < 0.001.

The basal levels of some molecular chaperones are decreased in cell models and the neuronal tissue of mouse models of HD. We wondered if the same phenomenon would hold true for peripheral tissues. To this end, we compared basal chaperone levels in neuronal tissue (cortex) with that in liver and two muscles throughout disease progression in the R6/2 mouse model^15^ (Fig. 2A). Expression levels in liver were largely unaffected at end stage disease (Fig. 2A; liver 14 wk), with slight changes at the pre-symptomatic stage (4 weeks). Intriguingly, in end stage muscles, most HSP genes were upregulated, in particular the *Hsp90* isoforms (Fig. 2A; quad. fem and tib. ant. 14 wk). One possible explanation for this observation is that muscle tissue, in contrast to neuronal tissue, has a higher proteostatic buffer capacity, allowing it to counteract the proteotoxic effects of mutant huntingtin to a larger extent and induce expression of molecular chaperones as a defensive strategy.

To investigate a possible impairment in the response to an *in vivo* heat shock in HD mouse models, we compared the expression levels of five HSP genes and *Hsf1* in 12 week old R6/2 mice with that in wild type littermates. As a control for chaperone gene induction, we used HSP90 inhibition (HSP990), which has been shown to result in an impaired HSR in the R6/2 mice^7^. In *quadriceps femoris*, both HS and HSP90 inhibition led to a significant induction of *Hspa1a/b*, *Dnajab1*, *Hspb1* and *Hsp90ab1* levels in both genotypes at 4 hours post treatment (Fig. 2B). In contrast, *Hsp90aa1* and *Hsf1* levels were unchanged (Fig. 2B). More importantly, as for HSP90 inhibition, the level of *Hspa1a/b*, *Dnajab1* and *Hspb1* induction by HS treatment was decreased in R6/2 mice (Fig. 2B; compare treated groups for HS and HSP990 and Figure S2). As for HSP990 treatment, the induction of *Hsp90ab1* was not impaired in R6/2 mice (Fig. 2B). We extended our analysis to another muscle (Figure S3A, *tibialis anterior*) and liver (Figure S3B), as well as cortex as an example of a neuronal tissue (Figure S3C). The expression of HSP genes was significantly induced through HS treatment in R6/2 tibialis compared to wild type, however, this was more variable than for quadriceps (Figure S3A). Variability was even higher in liver (Figure S3B). In most cases, we did not observe an impairment in chaperone gene expression in these two tissues, possibly as a consequence of this higher variability. Strikingly, chaperone expression was not induced in cortex by heat shock treatment (Figure S3C), despite the surface temperatures of the forehead and neck (Fig. 1D) being significantly increased. The heads had been excluded from the heat pad during the procedure, due to technical reasons involving the maintenance of anesthesia. We reasoned that quick heat dissipation through the fur could lead to a sub-threshold temperature in the brain for HSF1 activation. Indeed, the measurement of temperature within the brain with an implanted thermal probe revealed an increase in temperature of only about 2 °C (data not shown).

We analyzed chaperone protein levels 24 hours post treatment with heat shock (Fig. 2C) or HSP90 inhibition (Fig. 2D). In both cases, this led to significant increases in HSP70, HSP40 and HSP25 levels (Fig. 2C and D). When comparing the protein levels in 12 week old R6/2 mice with wild type mice, we observed an impairment in the induction of most HSP genes, consistent with the impairment at the mRNA level (Fig. 2C and D; compare HS or HSP990 treated R6/2 with HS or HSP990 treated wild type). Analysis of HSP protein levels in *tibialis anterior* (Figure S4A), liver (Figure S4B) and cortex (Figure S4C) largely mimicked the results obtained from their respective RNA analyses (Figure S3) as described above.

**Figure 3:**
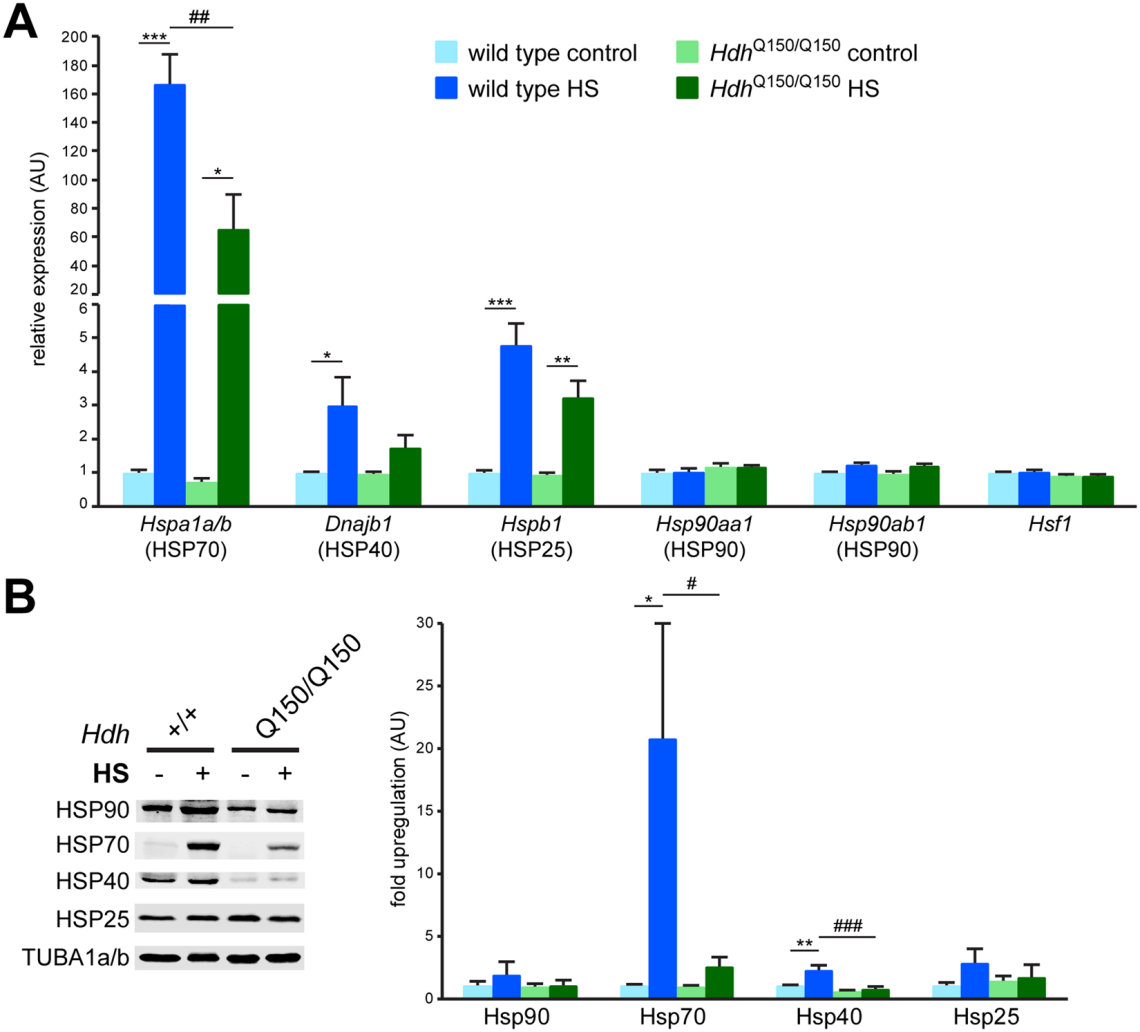
The heat shock response is impaired in the *Hdh*Q150 mouse model of Huntington’s disease. (**A**) Transcript induction at 4 hours after heat shock in *quadriceps femoris* muscle of homozygous *Hdh*Q150 and wild type mice at 21-22 months of age. Data are mean ± SEM relative to the levels of control wild type animals; n ≥ 5; two-way ANOVA with Tukey *post hoc* test. (**B**) Heat shock protein induction at 24 hours after heat shock in *quadriceps femoris* muscle of homozygous *Hdh*Q150 (Q150/Q150) and wild type (+/+) mice at 21–22 months of age. Data are mean ± SEM relative to the levels of control wild type animals; n ≥ 4; two-way ANOVA with Tukey *post hoc* test. TUBA1a/b was used as a loading control. Treatment: *p < 0.05, **p < 0.01, ***p < 0.001 and treatment/genotype ^#^ *p* < 0.05, ^##^ *p* < 0.01, ^###^ *p* < 0.001.

The R6/2 mouse model expresses exon 1 of mutant HTT and has an early onset and rapidly progressing phenotype. The *Hdh*Q150 mouse model expresses full length HTT with an expanded polyQ tract^16^, as well as fragments of HTT^17,18^ and develops very similar phenotypes to R6/2 mice, albeit with a later onset and slower rate of progression^19^. To ensure that the HSR impairment in R6/2 mice was not due to the very pronounced phenotype, we treated end stage (21-22 months) homozygote *Hdh*Q150 mice with our heat shock procedure (Fig. 3). Overall, the *quadriceps femoris* muscles of the *Hdh*Q150 mice showed the same pattern of impairment 4 hours post HS treatment when compared to wild type littermates (Fig. 3A and B).

### Transcriptome-wide analysis of HSR impairment in the R6/2 HD mouse model

Next, we analyzed the transcriptome-wide changes induced by heat shock (Table 1, S1 and S2) and HSP90 inhibition (Table 2, S3 and S4) in R6/2 mice and wild type littermates. We sequenced RNA from *quadriceps femoris* 4 hours post treatment, computed the significantly HS-regulated genes and performed gene ontology enrichment analysis. As expected, heat shock treatment in wild type mice led to a vast induction of genes involved in protein refolding (Table 1, wild type (WT); Figure S5A). Prediction of upstream regulators identified HSF1, validating our enrichment analysis pipeline. Interestingly, circadian locomotor output cycles kaput (CLOCK) transcription factor regulated genes were highly enriched in wild type mice, as well as in R6/2 (Table 1 and S1, column regulators). On the other hand, the HSR was severely blunted in R6/2 mice with only 109 genes regulated by HS (about 13% of WT) (Table 1, R6/2; Figure S5A). Additionally, HSF1 was not predicted to be a regulator for this response, again highlighting the impairment of HSF1 activation in R6/2 mice. Predicted regulators for the response in both R6/2 and wild type mice included kinases involved in immune system function (Table 1 and S1, e.g. IRAK4, ITK) and membrane bound growth factors receptors (Table 1 and S1, e.g. IGFR1, FGFR1, EGFR). Our prediction of chromatin modifications showed a high enrichment of heterochromatin associated marks like histone H3 lysine 9 tri-methyl (H3K9me3) and lysine 27 tri-methyl (H3K27me3) (Table 1; Figure S5B), which is consistent for genes, like molecular chaperones, that are repressed under normal conditions. We followed on by dissecting the common and specific (to WT or R6/2) responses to heat shock and could show that most of the genes that were changed in the R6/2 mice were also changed in wild type mice and only 22 genes were exclusively changed in the R6/2 mice (Table S1; Figure S5A). Due to this huge HSR impairment in R6/2 mice, and therefore the lack of a substantial R6/2 specific response, a transcription factor network could not be generated.

**Table 1:** Gene ontology enrichment analysis for heat shock treated mice.

**comparison**	**regulation**	**genes**	**regulators (1: ChEA, 2: ENCODE)**	**pathways (1: WIKIPathways, 2: Reactome)**	**gene ontologies (biological process)**	**kinases**	**chromatin marks**
WT control vs. HS	up	570	1: CLOCK 315.2, HSF1 302.42, ESR1 210.21, TCFAP2C 149.06, PPARG 143.32: MYOD1 55.7, MYOG 42.06, HSF1 41.33, EP300 34.79, ZMIZ1 34.45	1: Adipogenesis 23.72, Diurnally Regulated Genes with Circadian Orthologs 9.86, MAPK Signaling Pathway 8.47, Aryl Hydrocarbon Receptor 6.672: Attenuation phase (of HSF1) 26.5, HSF1-dependent transactivation 23.52, Cellular response to heat stress 16.7	response to topologically incorrect protein 39.17, protein refolding 28.18, regulation of p38MAPK cascade 21.76, cellular response to lipid 16.21, regulation of vasculature development 14.86	IRAK4 122.8ILK 83.21IGF1R 56.46ITK 42.54EGFR 41.07	H3K4me1 36.38H3K27me3 27.61H3K27ac 19.79
	down	264	1: WT1 86.33, NANOG 81.37, POU5F1 80.23, EP300 66.67, ZFP281 63.942: MAZ 14.9, EP300 12.0, GATA2 10.61, MYOD1 10.53, TCF3 9.86	1: not significant2: Signaling by BMP 13.73, Defective SLC26A2 causes chondrodysplasias 7.29, Extracellular matrix organization 7.19, Defective EXT1 causes exostoses 1, TRPS2 and CHDS 7.18, Oncogene Induced Senescence 7.11	cellular response to lipid 21.06, receptor protein serine/threonine kinase signaling pathway 18.85, BMP signaling pathway 18.08, positive regulation of endothelial cell migration 17.62, regulation of smooth muscle cell migration 15.15	IRAK4 41.39FGFR1 31.01CDK8 19.9BMPR2 10.99KSR2 8.7	H3K27me3 31.73H3K4me3 23.62H3K4me1 17.47
R6/2 control vs. HS	up	89	1: CLOCK 69.16, STAT3 45.91, ESR1 40.48, SUZ12 34.81, EGR1 33.552: not significant	1: Corticotropin-releasing hormone 7.05, AGE/RAGE pathway 6.15, Diurnally Regulated Genes with Circadian Orthologs 5.052: HSF1 activation 11.17, Attenuation phase (of HSF1) 11.09, HSF1-dependent transactivation 10.9, Cellular response to heat stress 8.24	cellular response to lipid 15.92, cellular response to hormone stimulus 13.81, negative regulation of calcium ion transport 11.38, response to temperature stimulus 11.12, nitric oxide mediated signal transduction 10.37	IRAK4 20.98AKT1 20.3RAF1 16.98CDK8 9.51ROCK1 8.63	H3K9me3 13.86H3K27me3 10.28H3K4me1 10.24
	down	20	1: EP300 19.44, KLF4 7.98, VDR 7.96, GATA2 7.00, DNAJC2 7.002: not significant	1: Striated Muscle Contraction 13.98, Retinol metabolism 8.342: Striated Muscle Contraction 8.19, Signaling by Retinoic Acid 7.79	actin-myosin filament sliding 17.2, negative regulation of cartilage development 13.33, response to leptin 5.41	FGFR1 6.61AKT1 5.56	not significant
*Hsf1* ^−/−^ control vs. HS	up	239	1: ESR1 134.55, CLOCK 108.64, STAT3 91.19, MTF2 80.56, ZNF217 76.022: MYOD1 29.47, ESR1 21.0, STAT3 18.65, TCF12 18.6, RAD21 18.36	1: Adipogenesis 14.47, Oncostatin M Signaling Pathway 9.54, Spinal Cord Injury 9.08, Diurnally Regulated Genes with Circadian Orthologs 8.872: not significant	response to oxygen levels 13.14, regulation of heart contraction 12.63, negative regulation of phosphorylation 11.29, negative regulation of cell migration 10.48, Rho protein signal transduction 10.29	IRAK4 70.63KSR1 35.67CDK8 27.78HUNK 23.99IGF1R 19.05	H3K4me1 28.94H3K27me3 24.01H3K9me3 22.37
	down	103	1: TCF3 41.25, PPARG 37.09, CLOCK 32.42, POU5F1 28.47, POU3F2 28.472: GATA2 5.63	1: Striated Muscle Contraction 17.28, PPAR signaling pathway 12.95, Adipogenesis genes 7.35, SIDS Susceptibility Pathways 5.782: Striated Muscle Contraction 14.29	muscle filament sliding 24.95, circulatory system process 11.09, positive regulation of osteoclast differentiation 9.22, fat cell differentiation 8.75, lipid storage 8.65	CDK8 24.31ILK 21.09FGFR1 20.89AKT2 12.6IRAK4 8.16	H3K27me3 19.97H3K9me3 13.08H3K9ac 7.42

Upstream regulators were predicted using the ChIP-x Enrichment Analysis (1: ChEA) and ENCODE transcription factor ChIP-seq database 2015 (2: ENCODE).v Chromatin marks were predicted using the ENCODE histone modifications database 2015. Only the top non-redundant significantly enriched terms followed by their combined score are shown. See also Tables S1, S2, S5, S6 and S8.

**Table 2:** Gene ontology enrichment analysis for HSP990 treated mice.

**comparison**	**regulation**	**genes**	**regulators (1: ChEA, 2: ENCODE)**	**pathways (1: WIKIPathways, 2: Reactome)**	**gene ontologies (biological process)**	**kinases**	**chromatin marks**
WTvehicle vs. HSP990	up	479	1: HSF1 382.44, ESR1 177.22, CLOCK 143.11, EP300 131.96, TCF21 130.52: HSF1 67.04, TCF12 56.90, MYOD1 53.53, GATA3 38.37, TCF3 36.89	1: Focal Adhesion 26.64, Inflammatory Response Pathway 16.87, IL-2 Signaling Pathway 9.78, Apoptosis-related network due to altered Notch3 in ovarian cancer 9.42, Type II interferon signaling 8.592: Extracellular matrix organization 61.98, Collagen biosynthesis and modifying enzymes 32.3, Attenuation phase (of HSF1) 25.24, HSF1 activation 24.29, Scavenging by Class A Receptors 12.71	extracellular matrix organization 96.51, collagen fibril organization 43.4, protein folding 43.24, response to wounding 23.12, response to acid chemical 19.93	IRAK4 158.7KSR1 83.98CDK8 72.45BMPR2 62.38FGFR1 42.72	H3K27me3 37.46H3K27ac 28.67H3K4me1 26.28
	down	195	1: CLOCK 95.44, TAF7L 90.88, SOX2 60.96, FOXP2 57.93, TCFAP2C 51.392: RFX5 18.54, POLR2A 13.64, E2F1 13.31, EP300 10.71, MAZ 10.24	1: not significant2: not significant	response to peptide 7.13, organic acid transport 6.77, cellular response to oxidative stress 6.68, positive regulation of p38MAPK cascade 6.62, negative regulation of lipid storage 5.48	ILK 24.46IRAK4 24.10IGF1R 16.38HUNK 14.21KSR2 13.27	H3K4me1 13.05H3K27me3 10.84H3K4me3 9.79
R6/2vehicle vs. HSP990	up	149	1: HSF1 232.59, CLOCK 51.98, TRIM28 36.67, SUZ12 35.25, E2F1 32.962: HSF1 53.66, STAT2 10.42, STAT1 10.03, EP300 9.31, FOSL1 9.24	1: IL-4 Signaling Pathway 6.42, Apoptosis Modulation and Signaling 6.14Adipogenesis 6.06, MAPK Signaling Pathway 5.35, SIDS Susceptibility Pathways 5.122: HSF1 activation 39.15, Attenuation phase (of HSF1) 39.11	protein folding 32.7, regulation of apoptotic signaling pathway 13.91, negative regulation of phosphorylation 12.27, regulation of cytokine production 11.32, response to interferon-alpha 8.11	CDK8 34.46ILK 20.06ROCK2 18.68IRAK4 13.52ROCK1 13.48	H3K4me3 13.07H3K4me1 8.03H4K20me1 6.73
	down	249	1: CLOCK 101.11, NANOG 80.97, ESR1 78.05, MTF2 70.52, PPARG 66.172: TCF12 15.54, CTCF 15.46, NFIC 14.83, ZC3H11A 14.13, UBTF 13.55	1: Adipogenesis 11.53, Signaling Pathways in Glioblastoma 7.63, EGF/EGFR Signaling Pathway 5.48, MAPK Signaling Pathway 5.472: Signaling by EGFR 6.47, Signalling by NGF 6.4, Downstream signaling of activated FGFR 6.18, Signaling by ERBB2 6.04, Signaling by FGFR 6.03	cellular response to nitrogen compound 15.21, regulation of vasculature development 13.83, positive regulation of lipase activity 13.57, Ras protein signal transduction 12.82, positive regulation of reactive oxygen species metabolic process 12.41	IGF1R 37.05KSR2 33.07IRAK4 31.62HUNK 24.12ROCK2 10.21	H3K27me3 38.44H3K4me1 33.46H3K9me3 12.79
*Hsf1* ^−/−^ vehicle vs. HSP990	up	72	1: TAF7L 47.7, ATF3 43.03, STAT3 42.39, SOX2 36.48, E2F1 35.422: CEBPB 47.9, CHD1 28.94, JUN 26.57, MAX 22.46, FOSL2 21.12	1: Hypertrophy Model 14.84, MAPK signaling pathway 6.282: PERK regulates gene expression 13.76, ATF4 activates genes 13.62, Amino acid transport across the plasma membrane 13.13, Amino acid synthesis and interconversion (transamination) 9.97, Unfolded Protein Response (UPR) 6.99	response to endoplasmic reticulum stress 17.21, response to unfolded protein 17.1, amino acid transmembrane transport 16.99, anion transmembrane transport 16.72, apoptotic signaling pathway 16.07	KSR1 38.98EGFR 30.53ALK 24.9IRAK4 21.98SNRK 16.38	H3K36me3 10.33H3K4me1 8.64H3K27me3 7.21
	down	40	1: KLF4/5/2 22.37, SMAD4 18.72, SMARCA4 18.57, SMAD3 16.92, ZFP281 15.52: not significant	1: Striated Muscle Contraction 8.062: Muscle contraction 10.87, Signaling by NOTCH4 5.39,	negative regulation of ERK1 and ERK2 cascade 9.92, actin-myosin filament sliding 9.45, negative regulation of intracellular signal transduction 9.41, cellular response to fluid shear stress 9.0, regulation of endothelial cell differentiation 8.77	KSR2 7.91	H3K27me3 6.32

Upstream regulators were predicted using the ChIP-x Enrichment Analysis (1: ChEA) and ENCODE transcription factor ChIP-seq database 2015 (2: ENCODE). Chromatin marks were predicted using the ENCODE histone modifications database 2015. Only the top non-redundant significantly enriched terms followed by their combined score are shown. See also Tables S3, S4, S5, S7 and S9.

Both HSP90 inhibition and heat shock led to a similar number of regulated genes in wild type animals (Table 1 and Table 2, WT). However, at the molecular level, the responses were considerably different (Figure S6 and Table S5). In summary, HSP90 inhibition led to a response with gene ontology terms (GO) that included: focal adhesion, extracellular matrix organization and angiogenesis, while heat shock was associated with higher levels of energy metabolism related genes (Table S5). Network analysis of predicted upstream regulators identified a set of transcription factors that were utilized by both stress responses (Figure S6C), including known regulators of the HSR like EP300^20^. In support of the GO enrichment analysis, we identified the GA binding protein transcription factor alpha subunit (GABPA, also known as NRF2), a transcription factor required for mitochondrial biogenesis^21^, as a heat shock specific transcription factor (Figure S6C).

**Figure 4:**
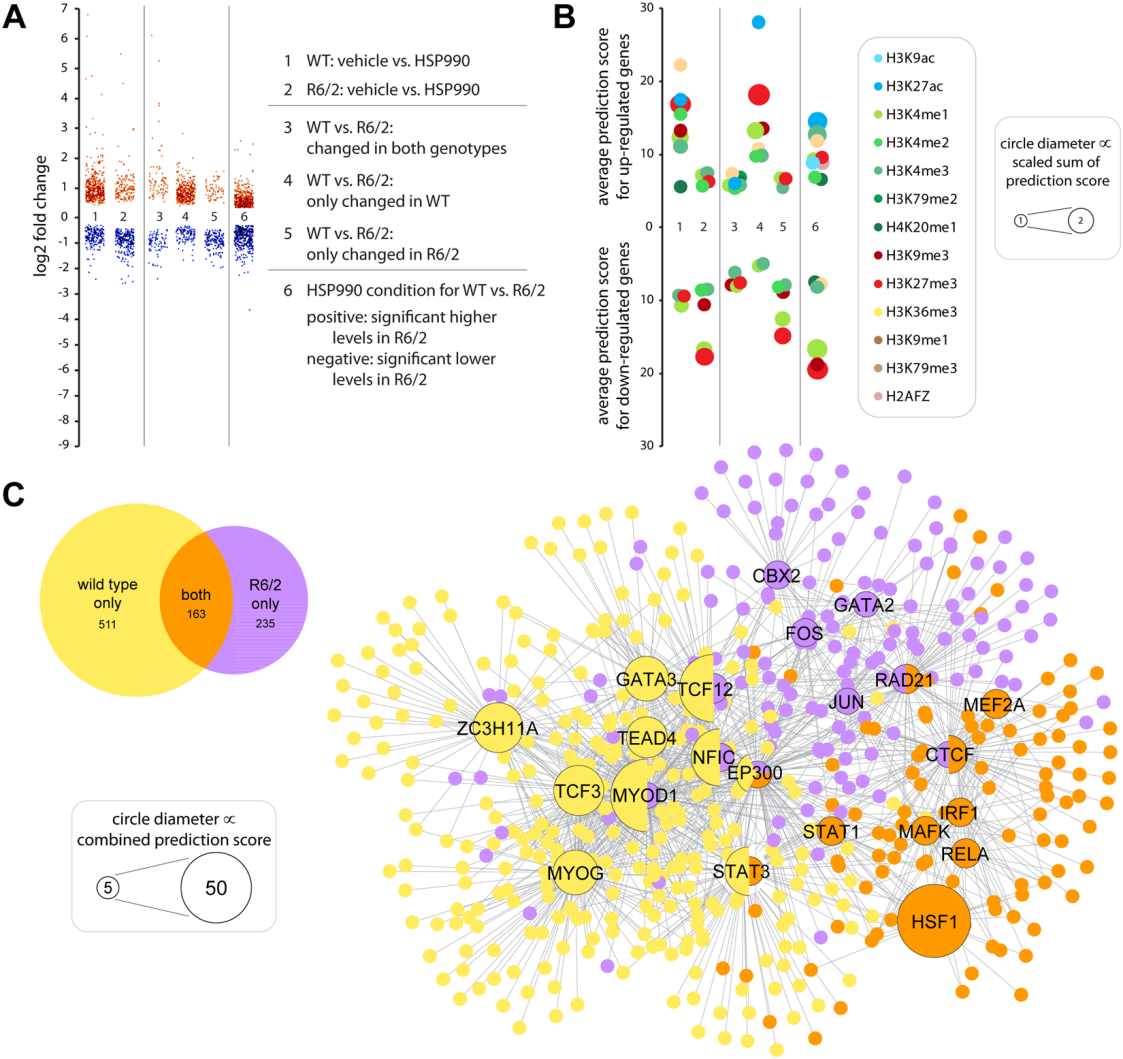
Differential systemic response to HSP90 inhibition in R6/2 compared to wild type mice. (**A**) Scatter plot showing the significant log_2_ fold changes at 4 hours after HSP90 inhibition (HSP990) in *quadriceps femoris* muscle of R6/2 and wild type mice at 12 week of age. Each dot represents a gene. Lanes 1 and 2 represent the significantly regulated genes through HSP90 inhibition in R6/2 or wild type mice. Lanes 3 to 5 show the common (lane 3) and distinct (lanes 4 and 5) responses to treatment. Lane 6 compares HSP990 treated R6/2 with HSP990 treated wild type mice. Here, we corrected for differences due to the genotype by subtracting the log_2_ fold changes of significantly different genes (genotype) from their log_2_ induction value (HSP990). Only genes with a resulting fold change of ≥1.25 were considered for further analysis. (B) Chromatin mark predictions for genes shown in (**A**). Only significantly enriched chromatin marks (*p* < 0.001) were considered. We used the combined score, which is the product of the *p*-value with the z-score of the deviation from the expected rank, as a measure for prediction quality. Together, the average (y-axis) and the sum (circle diameter) of the combined scores are a good indicator of the confidence of the chromatin mark predictions. (**C**) Venn diagram and transcription factor network of common and distinct responses to HSP90 inhibition in R6/2 and wild type mice. Data correspond to lanes 3, 4 and 5 in (**A**) and (**B**). Numbers indicate the number of significantly regulated genes. To predict upstream regulators, we created gene lists for significantly regulated (up and down combined) genes for each condition and used the ENCODE transcription factor ChIP-seq database (2015) to identify the significantly enriched transcription factors (n ≤ 10 with a combined score of ≥ 5). Circle diameter is an indicator of the confidence of the predictions. See also Figures S2, S5 and S6.

HSP90 inhibition in wild type mice predominantly led to an induction of genes involved in extracellular matrix and immune system related processes (Table 2, WT; see also Figure S6), in comparison to heat shock where the predominant GO term was protein folding. Remarkably, R6/2 mice showed a considerable transcriptional response to HSP90 inhibition, in contrast to the severely blunted response that had occurred following heat shock (Fig. 4A and Table 2, R6/2). To further analyze this, we again separated the response that occurred in both WT and R6/2 mice, from the genotype-specific genes sets (Fig. 4A and Table S3). The proteostasis component of the response was shared between WT and R6/2 (Table S3), although, the level of this response was impaired in the R6/2 mice (Table S4). On the other hand, the extracellular matrix component of the response was only enriched for WT mice (Table S3). The predominant R6/2-specific effect was a suppression of the immune system (Table S3). In response to HSP90 inhibition, our chromatin mark prediction analysis showed that up-regulated genes in wild type and downregulated genes in R6/2 mice were normally associated with heterochromatin (Fig. 4B). Our transcription factor networks (Fig. 4C) confirmed the GO enrichment analysis (Table S3) and showed that an HSF1-dependent response was shared between both genotypes. Moreover, in wild type mice HSP90 inhibition appeared to induce a similar program to ‘wounding’, leading to the activation of several muscle related transcription factors (Fig. 4C and Table S3, e.g. MYOG, MYOD1, TEAD4). Furthermore, when analyzing only the treated groups in R6/2 compared to wild type (see also Figure S2A, genotype and treatment effects), it was evident that in both cases, for heat shock (Table S2) and HSP90 inhibition (Table S4), proteostasis related genes were significantly more highly induced in wild type than in R6/2 mice. There was no significant GO term for genes that were more highly induced in R6/2 mice through heat shock (Table S2). Genes that were more highly induced in R6/2 mice through HSP90 inhibition were enriched for muscle function and energy metabolism related genes (Table S4).

### HSF1-dependent and –independent responses to heat shock and HSP90 inhibition

**Figure 5:**
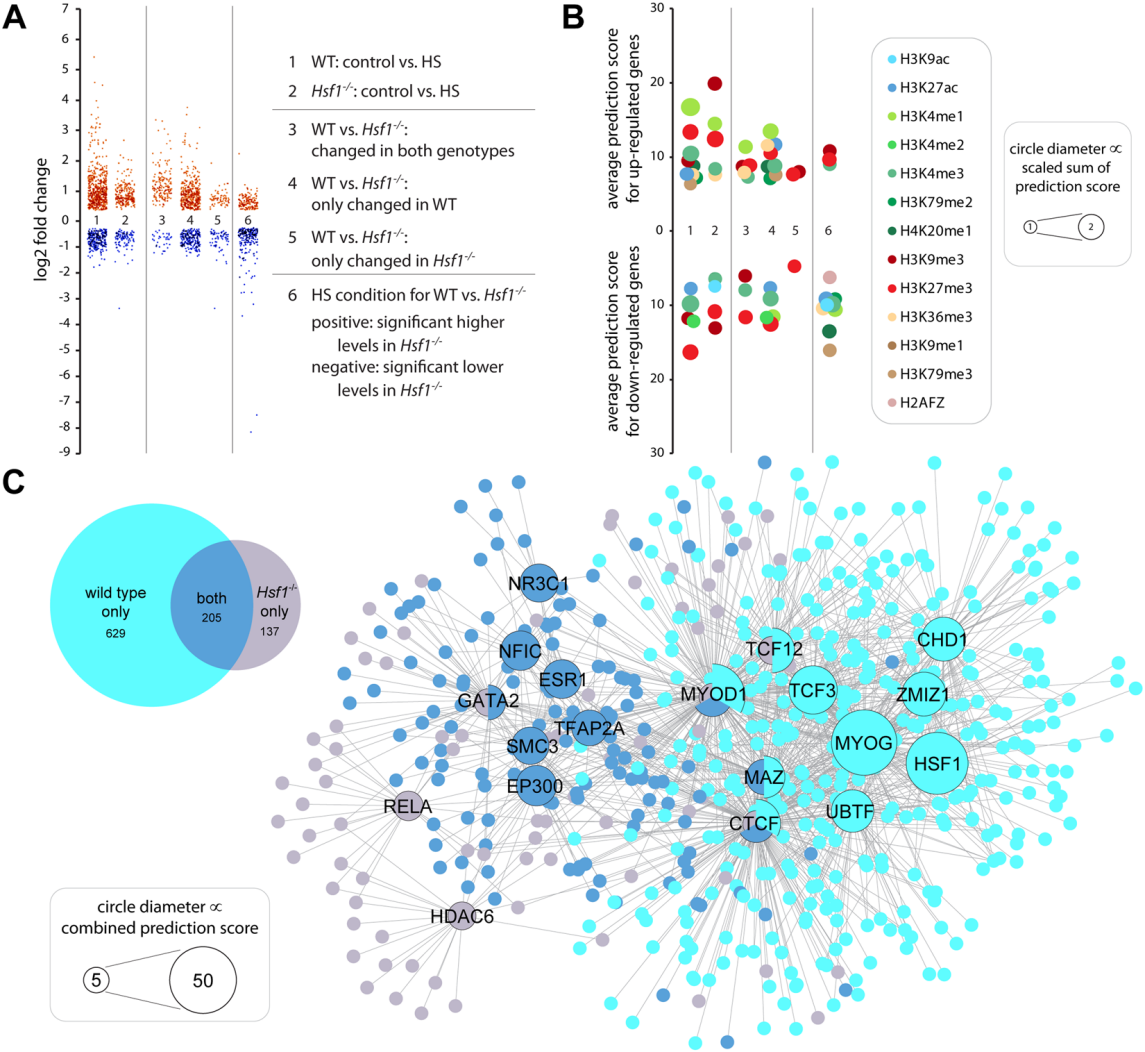
Differential systemic response to heat shock in *Hsf1* knockout compared to wild type mice. (**A**) Scatter plot as described in Fig. 4A showing the log_2_ fold transcriptome wide changes at 4 hours after heat shock (HS) in *quadriceps femoris* muscle of *Hsf1* knockout (*Hsf1* ^−/−^) and wild type mice at 10–12 week of age. (**B**) Chromatin mark predictions for genes shown in (**A**). Chromatin Marks prediction was as described in Fig. 4B. (**C**) Venn diagram and transcription factor network of common and distinct responses to heat shock in *Hsf1* knockout and wild type mice. Data correspond to lanes 3, 4 and 5 in (**A**) and (**B**). Upstream regulator prediction was as described in Fig. 4B.

To analyze the dependency and independency of HSR regulated genes on HSF1, we compared the HSR in homozygous *Hsf1* knockout mice (*Hsf1* ^−/−^) with the HSR in wild type mice. We again focused on *quadriceps femoris* muscle at 4 hours post treatment with heat shock. Transcriptome-wide analysis of heat shock responsive genes revealed that in the *Hsf1* knockout mice a large number of genes were significantly regulated (about 40% of those regulated in WT, Table 1 and Fig. 5A). Interestingly, this response was also more than 3 times larger than the response in R6/2 mice indicating that not only the regulation of HSF1 dependent genes is impaired in R6/2 (Table 1). The induced genes did not cluster in a particular pathway and were only enriched for those involved in muscle function (Table 1). Validating the analysis pipeline, HSF1 was not identified as a *bona fide* transcription factor. However, several transcription factors that were predicted to be upstream regulators in wild type, e.g. CLOCK, ESR1, MYOD1 etc., were again predicted as regulators in the *Hsf1* knockout mice (Table 1). The same was true for kinase prediction, for which a very similar set of kinases in wild type and *Hsf1* knockout mice was identified (Table 1). Dissecting the responses that were common to both wild type and *Hsf1* knockout mice and those specific to each genotype, we detected 137 genes that were only regulated in the *Hsf1* knockout mice (Fig. 5C and Table S6). These genes were enriched for those involved in muscle function, potentially regulated by the transcription factors identified in Fig. 5C. Next, we analyzed a distinct set of genes that was regulated in both genotypes independently of HSF1. Gene ontology enrichment analysis pointed towards activation of the p38/MAPK cascade (Fig. 5C and Table S6). Additionally, through our network analysis we were able to identify several potential regulators for this gene set including known HSR regulators like EP300 (Fig. 5C).

HSP90 inhibition resulted in only a small set of genes being significantly regulated in the *Hsf1* knockout mice (Table 2 and Figure S7A, *Hsf1* ^−/−^), which was mostly different to the response seen in wild type animals (Table S7). Gene ontology enrichment analysis indicated activation of the unfolded protein response (UPR) in the endoplasmic reticulum (Table 2 and Table S7). Consistent with this, transcription factors like ATF3 and CEBPB, which are both involved in the UPR, were predicted as upstream regulators for this gene set (Table S7 and Figure S7C).

The comparison of the treated (HS and HSP990) groups (see also Figure S2A, genotype and treatment effects) strengthened our findings that HSP induction *in vivo* was dependent on HSF1 (Figure S1 and Tables S8 and S9). Gene ontology enrichment showed significantly higher levels of protein folding related genes in wild type animals. Furthermore, HSF1 was predicted with very high scores as an upstream regulator for induced genes only in the wild type animals (Tables S8 and S9). Although we observed, for both HS and HSP90 inhibition, a set of genes that had higher levels in *Hsf1* knockout mice, it was not possible to assign them to a specific pathway. However, genes that were different between *Hsf1* knockout and wild type mice when treated with a heat shock could potentially be regulated by their chromatin state (Fig. 5B). In particular, the methylation status of histone H3 lysine 9 (mono-methyl or tri-methyl) was strongly associated with the gene sets of differentially regulated genes (Fig. 5B, lane 6).

## Discussion

The proteostasis networks can easily buffer diurnal changes in body temperature; these are comparably small, in the range of about one degree in humans. On the other hand, hyperthermia, which induces proteotoxic stress and can lead to severe problems, is mostly associated with prolonged exposure to high temperatures or with disease. In this study, we analyzed the mammalian heat shock response by inducing a very rapid *in vivo* heat shock in mice (41.5 °C ± 0.2 °C for 15 minutes), comparable to that which has been used in lower eukaryotes. We compared the HSR in the skeletal muscle of wild type mice with that of *Hsf1* knockouts and of mouse models of Huntington’s disease. We have defined the mammalian HSR *in vivo*, demonstrated that this is impaired in Huntington’s disease mouse models and identified HSF1-dependent and –independent regulated pathways.

We found that several hundred genes were regulated by heat shock in the muscle of wild type mice. The upregulated genes in this dataset were, as expected, enriched for genes involved in protein folding and with HSF1 predicted to be an upstream regulator (Table 1). Our data also indicated that the p38/MAPK pathway had been activated, possibly mediated by insulin like growth factor 1 receptor (IGF1R) and/or epidermal growth factor receptor (EGFR) (Table 1). Furthermore, we observed the modulation of a muscle function related program with induction of angiogenesis and regulation of cell migratory pathways. It has previously been reported that heat shock in a cell line leads to an activation of cytoskeleton related genes^12^. The authors speculate that the activation of these genes leads to the formation of stress fibers, which in turn activate a HSF1 independent transcriptional response. However, in this study we did not observe induction of cytoskeleton related pathways through heat shock (Table 1), nor through HSP90 inhibition (Table 2) indicating differential regulation of the HSR in single cells versus cells in a tissue context.

It has been reported that there is a general transcriptional repression in response to heat shock. The number of transcriptionally repressed genes after heat shock in mammalian cell lines ranges from several thousands to only a few hundred^12^. We observed significant (Benjamini-Hochberg corrected *p*-values < 0.05; no fold cut-off) repression of 264 genes at 4 hours post heat shock (Table 1) and 195 genes 4 hours post HSP90 inhibition (Table 2), which is less than for most previously reported studies. Methodological differences and slightly different time points after heat shock could account for some of this discrepancy. If this was the case, the discrepancies in the numbers of induced and repressed genes might be expected to be similar. However, the number of repressed genes in our dataset compared to previous studies is much lower, pointing towards a specific inhibition of certain pathways in the mammalian *in vivo* HSR, rather than a global inhibition of transcription.

Analysis of chromatin marks identified H3K27me3 as one of the major marks associated with heat shock regulated genes (Table 1; Figure S5B). H3K27me3 is a marker for repressed genes in heterochromatin domains^22^. This is consistent with the heat shock induced transcriptional activation of genes that are normally in an inactive state. Moreover, it has recently been shown that an increase of H3K27me3 marks at stress response gene loci is responsible for repression of the HSR in *C. elegans* during aging^13^. The authors found that reduction in a demethylase coincided with increased levels of histone H3 tri-methylation of K27 during aging in *C. elegans* and led to a diminished HSR. We also identified chromatin marks like H3K4me1, H3K4me3 and K3K27ac associated with actively transcribed genes (Table 1; Figure S5B)^22^. This chromatin signature is, amongst others, likely linked to circadian rhythm related genes as we observed induction of diurnally regulated genes and identified CLOCK transcription factor as an upstream regulator (Table 1). The small diurnal changes in body temperature in mammals usually correlate with circadian rhythms^23^. Intriguingly, changes in ambient or body temperature can reset circadian oscillators^24^. Additionally, heat stress^25^ and reactive oxygen species^26^ have been shown to reset circadian clocks in mouse fibroblast cell lines. Our analysis of the *in vivo* mammalian HSR indicates that this phenomenon can be translated onto an organismal level.

We used the ENCODE databases to predict upstream regulatory factors of the HSR and many of these transcription factors are known to interact with HSPs^27^. Binding of HSPs to HSF1 keeps it in an inactive state in the cytoplasm^28^. It is tempting to speculate that this interaction presents a similar regulatory mechanism for the inactivation of these transcription factors. Upon proteotoxic stress, chaperones bind to misfolded proteins, releasing the transcription factors allowing them to initiate a transcriptional response.

Huntington’s disease is caused by the expansion of the polyglutamine tract in the huntingtin protein (HTT), which leads to the abnormal folding of HTT and the formation of proteinaceous aggregates^29^. These mis-folded proteins should be recognized by the proteostasis network and molecular chaperones should be induced to aid the correct folding of the disease causing protein. While molecular chaperones do indeed recognize mutated HTT^30^, HSPs are not induced in severely affected neuronal tissue such as the striatum or cortex in human postmortem brain or mouse models. On the contrary, there is a progressive decline in the levels of several HSPs^31,32^ (see also Fig. 2A, cortex). By contrast, some neuronal tissues, such as the cerebellum, as well as peripheral tissues are in general less affected in HD. Recently, gene expression correlation network analysis revealed a group of proteostasis related genes that were upregulated in cerebellar *post-mortem* brain tissue from HD patients^33^. One possible explanation for this could be that these tissues are able to induce the proteostasis network and counteract the proteotoxic effects of mutant HTT to a certain extent. Supporting this, we observed induction of a subset of molecular chaperones in end-stage disease muscles (Fig. 2A). To the best of our knowledge, this is the first data showing that the proteostasis network is induced in peripheral mouse tissue through expression of mutant HTT. These findings also imply other, unknown mechanisms that suppress the basal induction in neuronal tissue.

Expression of mutant HTT negatively affects the molecular response to several stresses. This impairment has been described in various cell models and lower eukaryotes^34^, but there are only limited data available for the same phenomenon in mammals^7^. In this study we analyzed the HSR after heat shock or HSP90 inhibition in the skeletal muscle of two HD mouse models (R6/2 and *Hdh*Q150) compared with the HSR in wild type mice. Skeletal muscle atrophy and weakness is a well-documented feature of Huntington’s disease^35^, and HTT aggregation occurs in the skeletal muscle of both the R6/2 transgenic and *Hdh*Q150 knock-in models^36,37^. Analysis of the transcript levels of a set of molecular chaperones in both mouse models showed that the induction of these chaperones by both types of stresses was impaired on the transcript and protein level (Figs 2, 3 and S4). Our transcriptome-wide analysis of the HSR in the R6/2 HD mouse model supported these findings (Tables 1 and 2, Fig. 4 and S5). Furthermore, we could show that a heat shock led to a very minimal HSR with little over 100 genes regulated in the R6/2 mice (Table 1 and Figure S5). The response after a heat shock compared to that after HSP90 inhibition was, apart from the induction of molecular chaperones, very distinct (Figure S6 and Table S5). The fact that HSP90 inhibition mounted a considerable response in the R6/2 mice implies that HSP90 inhibition modulates pathways that are not impaired in HD.

We compared the HSR in muscle from wild type and *Hsf1* knockout mice and found no difference in the basal expression level of HSP genes (Figure S1). Previous studies have suggested that the expression of several HSP genes might be regulated by the interplay of multiple transcription factors, e.g. members of the MAF family, PAX6, NF-Y, NF-κB, or CREB^38,39^. Therefore, such a transcription factor interplay was sufficient for the basal expression of HSPs. In contrast, under heat shock conditions, mammalian HSF1 was essential for the induction of HSP genes (Figure S1). Although the expression of molecular chaperones was clearly dependent on HSF1, *Hsf1* knockout animals tolerated an *in vivo* heat shock very well for at least 24 hours, without any obvious adverse phenotypes. In contrast, a large percentage of *Hsf1* knockout animals did not tolerate HSP90 inhibition, some died overnight and the others were found unresponsive and were euthanized. A recent publication analyzing the HSR in the yeast *S. cerevisiae* found that the majority of the HSR was independent of Hsf1, which was only required to drive the expression of 9 genes after heat shock^11^. The authors proposed that the Hsf1 independent response could be driven by the yeast specific Msn2 and Msn4 transcription factors. In our analysis of the transcriptome wide HSR induced by an *in vivo* heat shock, we observed a significant regulation of several hundred genes in *Hsf1* knockout animals (Table 1). This response overlapped partly with the HSR in wild type animals, but the regulation of 137 genes was specific to *Hsf1* knockout mice (Table S6). The predicted regulation of this gene set (Fig. 5 and Table S6) by the NF-kB subunit RELA proto-oncogene (RELA) could represent a compensatory mechanism in the *Hsf1* knockout mice to upregulate pro-survival and proliferation genes^40^. Regulation by histone deacetylase 6 (HDAC6), which is a known modulator of the HSR by regulating the acetylation status of HSP90^41^ and is involved in stress granule and aggresome formation^42,43^, might contribute to the cellular stress response.

Intriguingly, the modulation of the circadian rhythm regulated genes appears to be independent of HSF1 and the CLOCK transcription factor was predicted as an upstream regulator in the *Hsf1* knockout animals (Table 1). These findings imply that the resetting of mammalian circadian clocks by heat shock is mostly independent of HSF1. Furthermore, previous findings have shown that binding of HSF1 to HSP gene promoters is diurnally regulated and HSF1 deficient mice exhibit alterations in circadian behavior^44^. Disruption of circadian rhythms has also been reported for HD patients and in HD mouse models^45^ highlighting the fact that pathways of the HSR and circadian clocks are tightly intertwined and dysregulated in Huntington’s disease.

## Methods

### Mouse maintenance, breeding, genotyping, and CAG repeat sizing

All experimental procedures performed on mice were approved by the University College London Ethical Review Process Committee and carried out under a Home Office License. *Hsf1* knockout mice were housed and genotyped as described^1^. R6/2 mice were housed and genotyped as described^46^. *Hdh*Q150 mice were housed and genotyped as described^18^. The mean CAG repeat size ± SD for all mice used in the entire study was 206 ± 5 for R6/2 mice and 167 ± 4 for the shorter and 187 ± 8 for the longer allele in the homozygous *Hdh*Q150 mice. Mice were culled by cervical dislocation, tissues were snap-frozen in liquid nitrogen and stored at −80 °C until further analysis.

### Heat shock treatment

Animals subjected to heat shock were anesthetized with isoflurane (Merial Animal Health Ltd.) with an oxygen flow of 2 l/min (Vaporiser IsoFlo Series 5, T.C.V BME; Vet Tech Solutions) and the whole body excluding the head was wrapped in a heating pad (CWE TC-1000; Linton Instrumentation) set at 41.5 °C ± 0.2 °C. Warm up period was in the range of 5 to 6 minutes to reach 41.5 °C. During heat shock the core body temperature was maintained at 41.5 °C ± 0.2 °C for 15 minutes, and was constantly monitored by a rectal probe. Following heat shock, animals were cooled, revived, and returned to their home cage. Control animals were treated in the same way, but core body temperature was maintained for 21 minutes at 36.9 °C ± 0.2 °C (equivalent to warm up period and 15 minutes of heat shock). Thermal camera images were captured using a U5855A TrueIR Thermal Imager, 350 °C (Keysight, Agilent). We did not find any gender specific effects when comparing female and male mice in the different treatment groups (two-way ANOVA, Bonferroni *post hoc* test p > 0.05 on the 4 hours post treatment datasets).

### HSP990 treatment

NVP-HSP990 (2-amino-7,8-dihdro-6H-pyrido[4,3-d]pyrimidin-5-one) was prepared and administered as described before^1^. Mice were dosed with 12 mg/kg HSP990 throughout this study. Heat shock treatment and HSP990 treatment were both conducted at the same time of the day (9 am to 12 pm) throughout this study.

### Quantitative PCR

RNA was extracted using QIAZOL together with RNeasy Mini kits (Qiagen) according to the manufacturer’s instructions. Reverse Transcription, Taqman RT quantitative PCR (qPCR) and the evaluation of the data was performed as previously described^1^. The geometric mean of the expression levels of the following housekeeping genes was used to standardize the samples: Figs 1E, 2A quad. fem., 2A tib. ant, 3A, S1A: *Atp5b*, *Sdha*, *Actb*; Fig. 2A cortex: *Atp5b*, *Sdha*, *Actb, Canx*; Fig. 2A liver: *Sdha*, *Canx*, *Ywhaz*; Fig. 2B: *Atp5b*, *Sdha*, *Actb*, *Ywhaz*; Figures S1B, S3A, S3C: *Atp5b*, *Sdha*; Figure S3B: *Atp5b*, *Sdha*, *Actb, Ubc*; Figure S3C: *Atp5b*, *Sdha*, *Gapdh*. Primers and probe sequences can be found in Table S10 of the supporting information.

### Antibodies and western blotting

Protein extraction and western blotting was carried out as described^1^. Blocking buffer was 5% (w/v) skimmed milk powder in TBS-T (50 mM Tris-Cl pH 7.4, 150 mM NaCl, 0.1% (w/v) Tween 20). All blots were incubated with primary antibodies over night at 4 °C in TBS-T. Wash buffer was TBS-T. Secondary antibodies were purchased from LI-COR and western blots were visualized on an Odyssey Sa (LI-COR) and analyzed with the Image Studio Lite Ver 3.1 (LI-COR). Antibodies and dilutions can be found in Table S11 of the supporting information.

### RNA sequencing

Samples were prepared using a modified strand-specific version of the Illumina Tru-Seq protocol. Final PCR amplification was performed with KAPA HiFi polymerase and GC buffer (KAPABIOSYSTEMS). The paired-end, strand-specific cDNA libraries were multiplexed onto the Illumina HiSeq (40 bp reads). Read data was mapped to the mm9 build with the Bowtie alignment program using setting –Best. 2 mice per treatment and genotype were sequenced (total n = 24). We compared the RNA sequencing data of a set of genes (HSPs, *Hsf1*, housekeeping genes) with qPCR data for a larger cohort (n ≥ 8) of mice to ensure that the mice that were used for RNA sequencing were a good representation of the average mouse.

### Bioinformatics

We used gene counts to compute estimated fold changes in expression levels with the DEseq. 2 pipeline^47^. We filtered genes that did not have equal or more than 2 counts in at least 10 samples across all sequenced samples. Custom gene lists for each condition and comparison were created by extracting significantly dysregulated genes (Benjamini-Hochberg corrected *p*-values < 0.05). We used Enrichr^48^ to conduct the subsequent gene ontology enrichment analysis for these lists. Upstream regulators were predicted using the ChIP-x Enrichment Analysis (ChEA) and ENCODE transcription factor ChIP-seq database 2015 (in Tables 1 to 10 labeled as 1: ChEA; 2: ENCODE). Chromatin marks were predicted using the ENCODE histone modifications database 2015. Only the most highly significantly enriched non-redundant (similar sub-terms were summarized into an overarching term) terms followed by their combined score are shown.

### Statistical analysis

All data were screened for outliers using a Grubbs’ test (GraphPad). All data were analyzed with IBM SPSS using a two-tailed homoscedastic Student’s *t*-test or a two-way ANOVA with either Tukey or Bonferroni *post hoc* test as specified in the Figure legends. Treatment: *p < 0.05, **p < 0.01, ***p < 0.001 and treatment/genotype ^#^ *p* < 0.05, ^##^ *p* < 0.01, ^###^ *p* < 0.001.

### Data availability

The datasets generated during and/or analysed during the current study are available in the Gene Expression Omnibus (GEO) repository, accession number GSE95602. https://www.ncbi.nlm.nih.gov/geo/query/acc.cgi?acc=GSE95602.

## Electronic supplementary material

Supplementary Info
