# Supplementary material for: HSF1-dependent and -independent regulation of the mammalian in vivo heat shock response and its impairment in Huntington's disease mouse models
Source: Sci Rep. 2017 Oct 2;7:12556. doi: 10.1038/s41598-017-12897-0 (PMC5624871; doi:10.1038/s41598-017-12897-0)
Supplement: Supplementary file 1 — Supplementary Info [file 41598_2017_12897_MOESM1_ESM.pdf]

SUPPLEMENTARY INFORMATION

HSF1-dependent and -independent regulation of the mammalian *in vivo* heat shock response and its impairment in Huntington's disease mouse models

Andreas Neueder[1]*, Theresa A. Gipson[2], Sophie Batterton[1], Hayley J. Lazell[1], Pamela P. Farshim[1], Paolo Paganetti[3,4], David E. Housman[2], Gillian P. Bates[1]*

Supplementary Figures

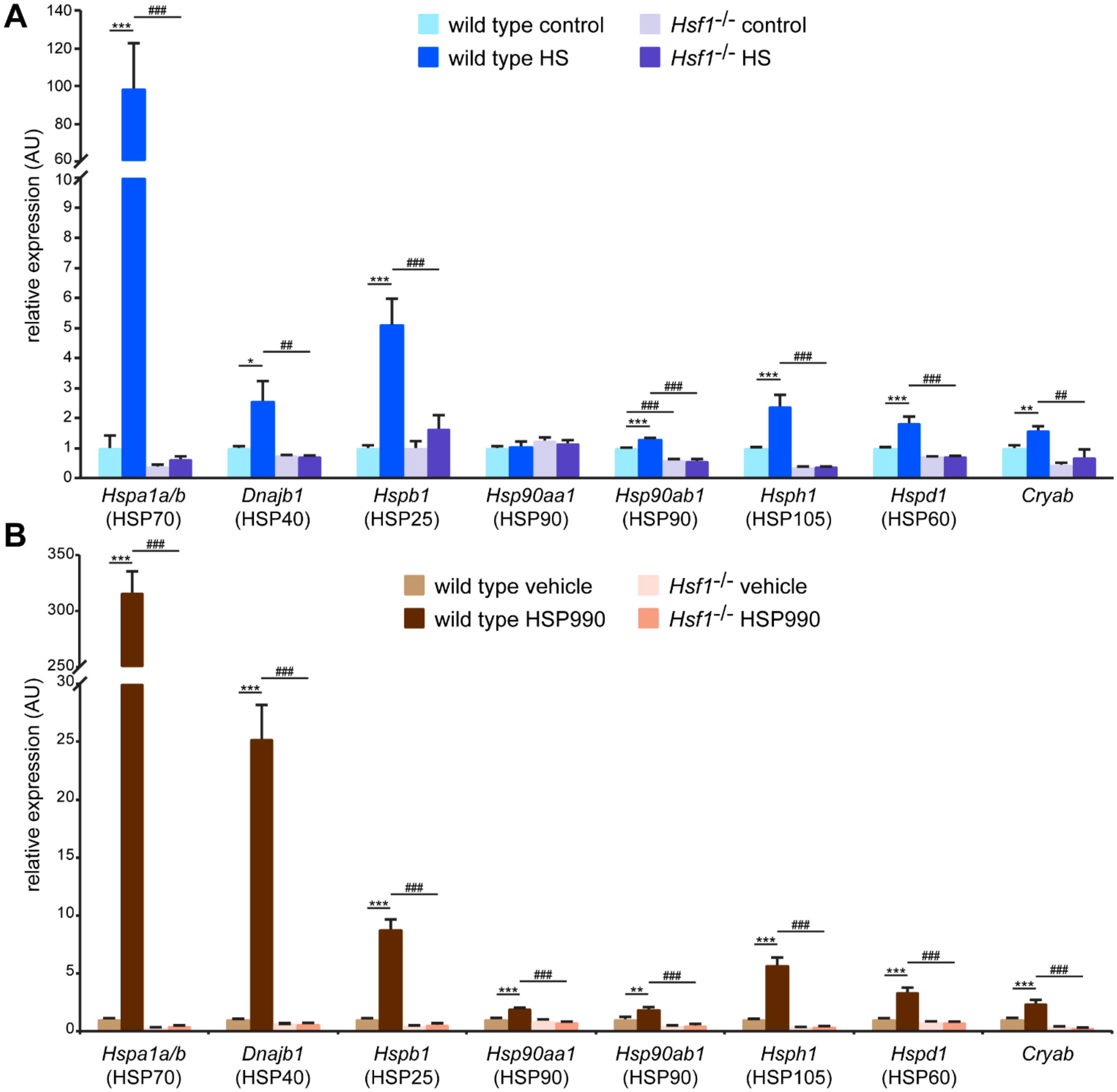

Figure S1. HSF1 dependent induction of HSP genes by heat shock or HSP90 inhibition.

Analysis of mRNA levels of HSP genes 4 hours after heat shock (HS) (A) or HSP90 inhibition (HSP990) (B) in *quadriceps femoris* muscle of *Hsf1* knockout mice (*Hsf1*$^{-/-}$) and wild type mice at 10-12 weeks of age. Data are mean ± SEM relative to the levels of control or vehicle treated wild type animals; n ≥ 6; two-way ANOVA with Tukey *post hoc* test. Treatment: *$p < 0.05$, **$p < 0.01$, ***$p < 0.001$; genotype/treatment: $^{\#\#}p < 0.01$, $^{\#\#\#}p < 0.001$.

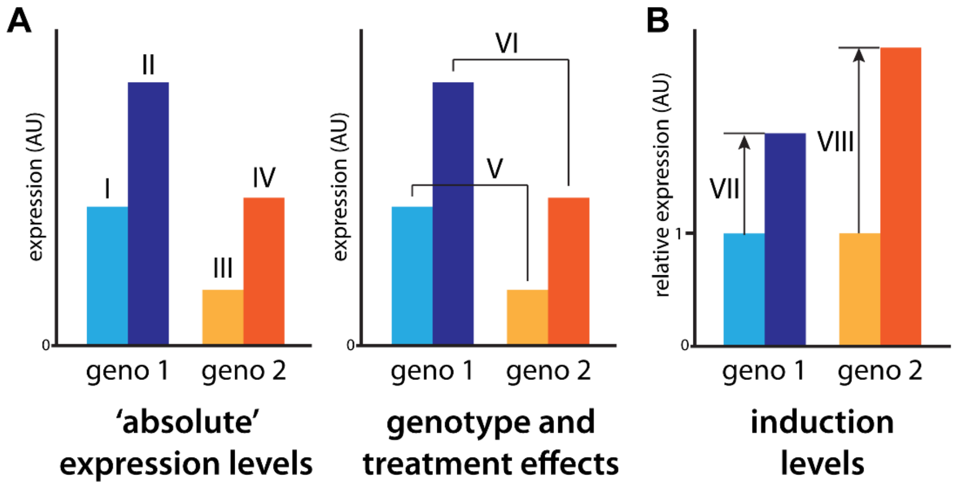

Figure S2. Schematic depicting the comparisons of treatment and genotype in this study.

(A) Comparison of the 'absolute' values of the expression of gene of interest (GOI). Left panel: The 'absolute' levels are standardized to housekeeping genes, and groups are all normalized to the same value (usually control/vehicle treated wild type). This allows a comparison of treatment effects (levels of II against I and IV against III), as well as analysis of effects due to genotype/treatment differences (right panel: V: un-treated condition = differences purely due to genotype; VI: treated conditions = differences due to genotype/treatment). This kind of comparison is used in Figure 2B, 2C, 2D; Figure 3A, 3B; Figure S1A, S1B, Figure S3A-C; Figure S4A-F. (B) Comparison of the levels of induction of the expression of GOI. The treatment groups are individually normalized to their respective control for each genotype. This analysis does not allow the absolute levels due to both treatment and genotype effects to be compared. Instead, it permits the genotype differences in response to treatment to be more easily compared. This kind of comparison is used in Figure 4 and 5; Figures S6 and S7. (A) and (B) the GOI levels can of course be lower in the treated groups, reversed for one genotype, or a combination of these. For better tangibility only upregulation of GOI levels due to treatment is shown in the figure.

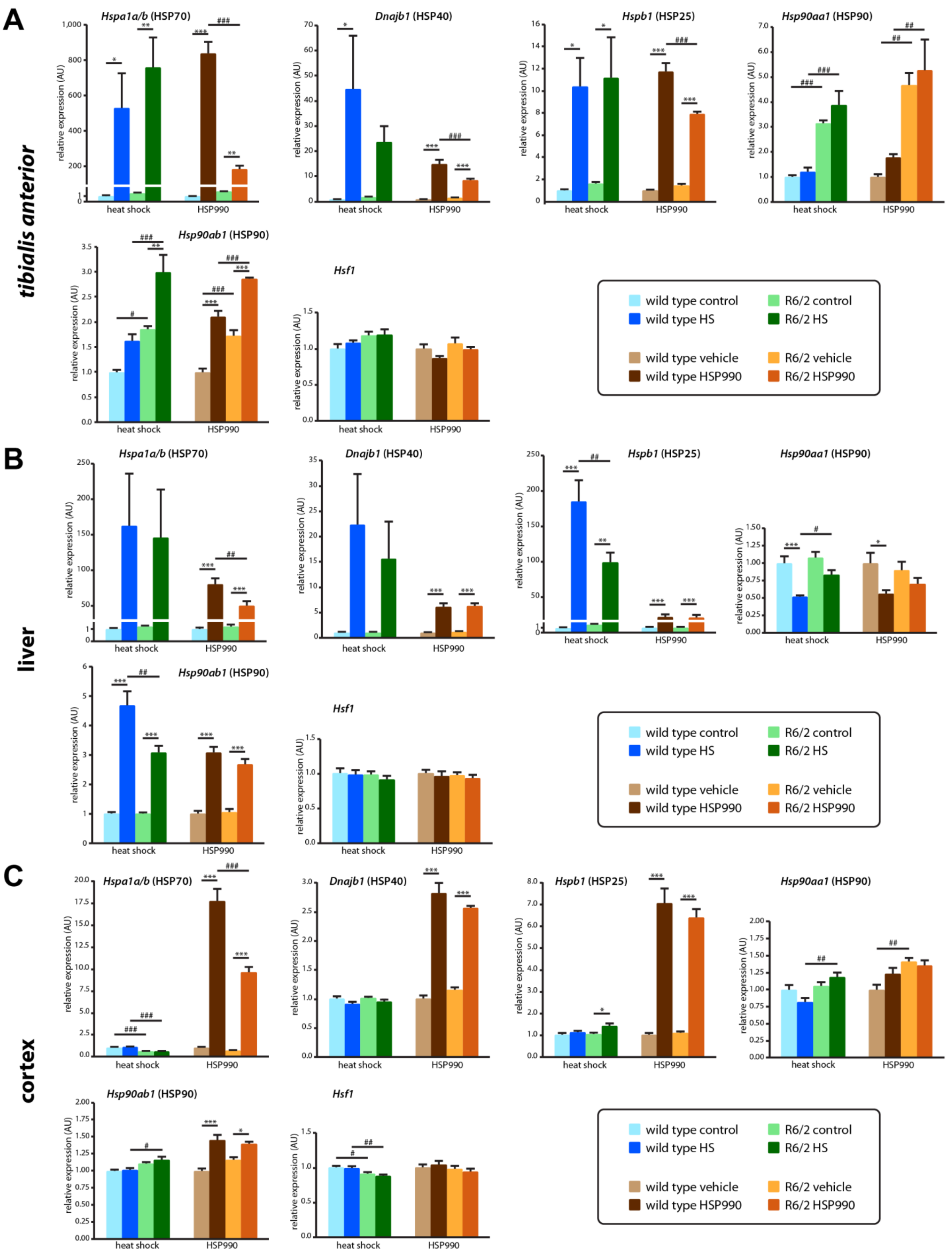

Figure S3. The heat shock response is impaired in the R6/2 HD mouse model at transcript level.

Transcript induction of heat shock response genes and *Hsf1* at 4 hours after heat shock (HS) or HSP90 inhibition (HSP990) in *tibialis anterior* muscle (A), liver (B) and cortex (C) of R6/2 and wild type mice at 12 week of age. Data are mean ± SEM relative to the levels of control or vehicle treated wild type animals; n ≥ 6; two-way ANOVA with Tukey *post hoc* test. Treatment: $^{*}p < 0.05$, $^{**}p < 0.01$, $^{***}p < 0.001$; genotype/treatment: $^{\#}p < 0.05$, $^{\#\#}p < 0.01$, $^{\#\#\#}p < 0.001$.

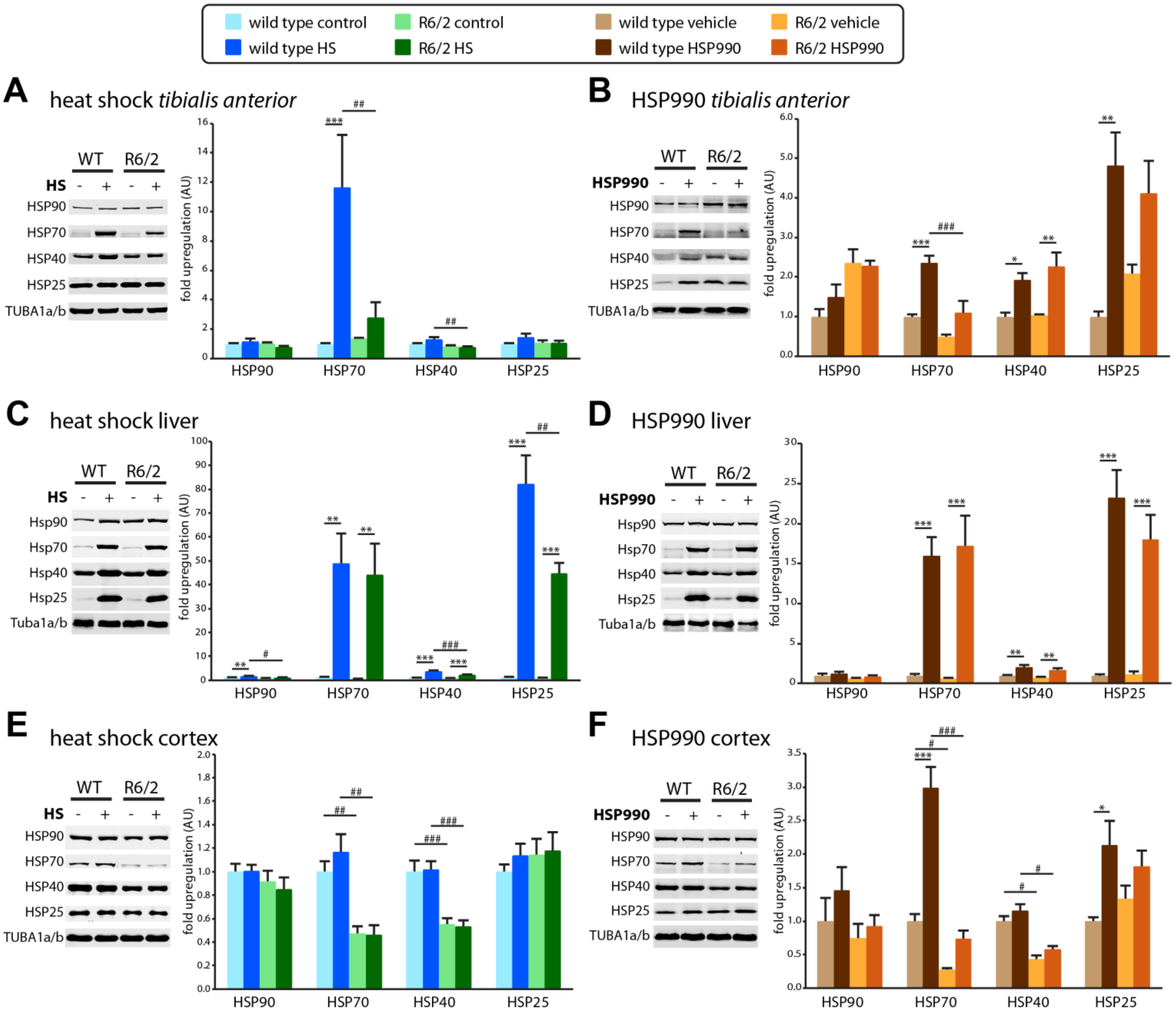

Figure S4. The heat shock response is impaired in the R6/2 HD mouse model at the protein level.

Heat shock protein induction (HSP90, HSP70, HSP40, HSP25) at 24 hours after heat shock (HS) or HSP90 inhibition (HSP990) of R6/2 and wild type mice at 12 week of age. Data are mean ± SEM relative to the levels of control or vehicle treated wild type animals; n ≥ 6; two-way ANOVA with Tukey *post hoc* test. Treatment: **p* < 0.05, ***p* < 0.01, ****p* < 0.001; genotype/treatment: $^{\#}p < 0.05$, $^{\#\#}p < 0.01$, $^{\#\#\#}p < 0.001$. TUBA1a/b was used as a loading control. (A) Heat shock treatment (HS) in *tibialis anterior* muscle. (B) HSP90 inhibition (HSP990) in *tibialis anterior* muscle. (C) Heat shock treatment (HS) in liver. (D) HSP90 inhibition (HSP990) in liver. (E) Heat shock treatment (HS) in cortex. (F) HSP90 inhibition (HSP990) in cortex.

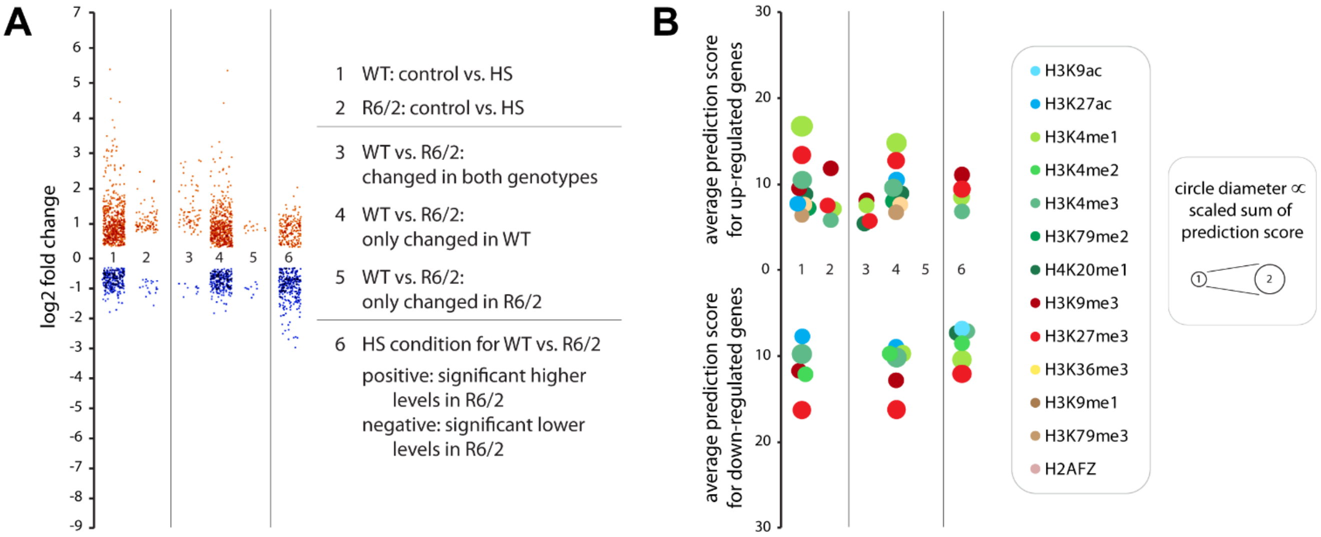

Figure S5. Differential systemic response to heat shock in R6/2 compared to wild type mice.

(A) Scatter plot showing the significant $\log_2$ fold transcriptome wide changes at 4 hours after heat shock (HS) in *quadriceps femoris* muscle of R6/2 and wild type mice at 12 week of age. Lanes 1 and 2 represent the significantly regulated genes through treatment with HS in R6/2 or wild type mice. Lanes 3 to 5 show the common (lane 3) and distinct (lanes 4 and 5) responses to treatment. Lane 6 compares HS treated R6/2 or wild type mice. Here, we corrected for differences due to the genotype by subtracting the $\log_2$ fold changes of significantly different genes (genotype) from their $\log_2$ induction value (HS). Only genes with a resulting fold change of ≥ 1.25 were considered for further analysis. (B) Chromatin mark predictions for genes shown in (A). Only significantly enriched chromatin marks ($p < 0.001$) were considered. We used the combined score, which is the product of the p-value with the z-score of the deviation from the expected rank, as a measure for prediction quality. Together, the average (y-axis) and the sum (circle diameter) of the combined scores are a good indicator of the confidence of the chromatin mark predictions.

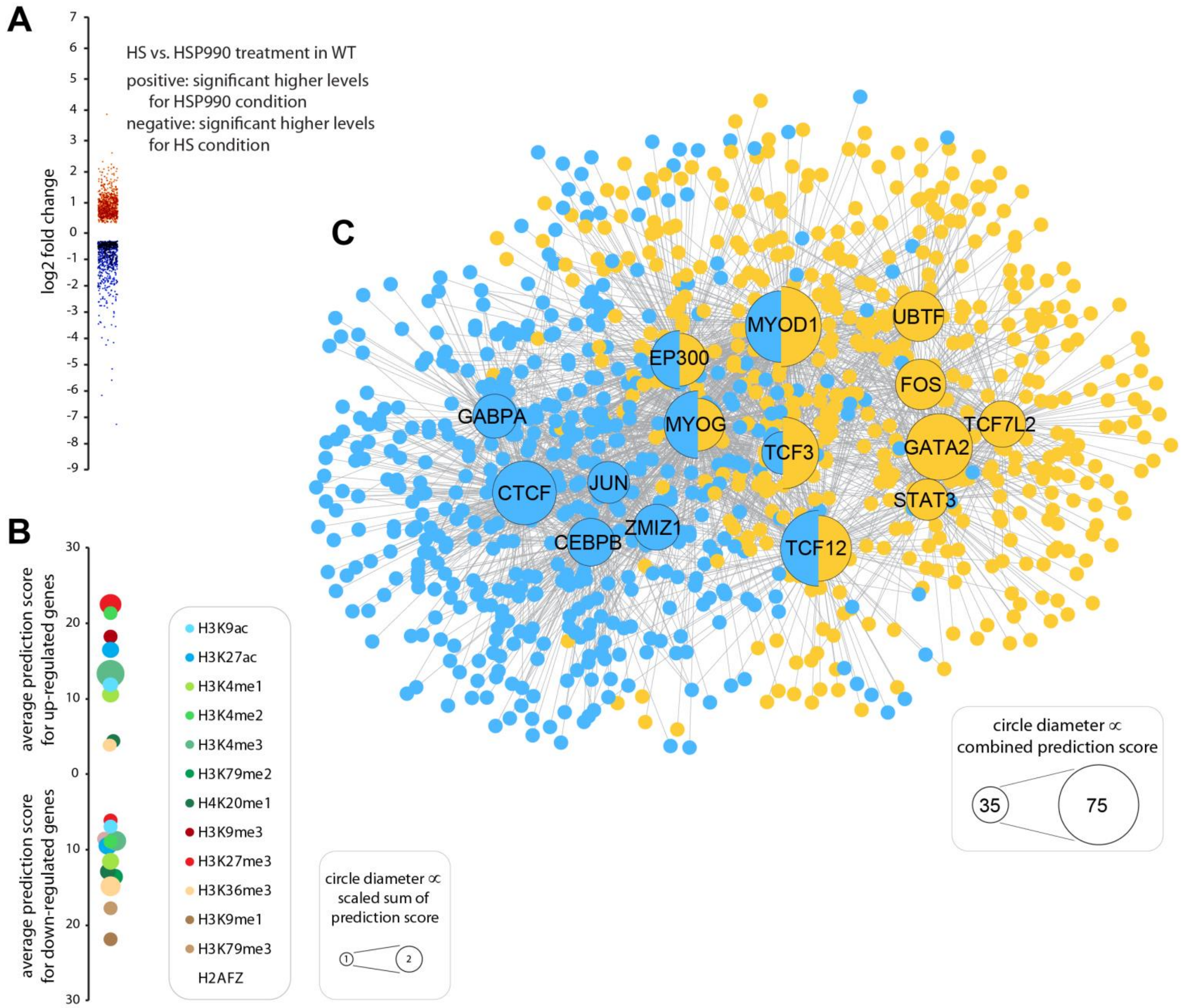

Figure S6. Comparison of the transcriptional response to heat shock and HSP990 in wild type mice.

(A) Scatter plot showing the significant $\log_2$ fold transcriptome wide changes at 4 hours after HSP90 inhibition (HSP990) and heat shock treatment (HS) in *quadriceps femoris muscle* of wild type mice at 12 week of age. We corrected for differences due to the effects of anesthesia (control) and vehicle treatment by subtracting the $\log_2$ fold changes of significantly different genes (vehicle vs. control) from their $\log_2$ induction value (HS or HSP990). Only genes with a resulting fold change of ≥ 1.25 were considered for further analysis. (B) Chromatin mark predictions for genes shown in (A). Only significantly enriched chromatin marks ($p < 0.001$) were considered. We used the combined score, which is the product of the p-value with the z-score of the deviation from the expected rank, as a measure for prediction quality. Together, the average (y-axis) and the sum (circle diameter) of the combined scores are a good indicator of the confidence in the chromatin mark predictions. (C) Transcription factor network of responses to heat shock (HS) and HSP90 inhibition (HSP990) in wild type mice. 719 dysregulated genes were significantly higher induced by HS and 747 genes by HSP990 treatment. To predict upstream regulators, we created gene lists for these significantly regulated (up and down combined) genes for each condition and used the ENCODE transcription factor ChIP-seq database (2015) to identify the significantly enriched transcription factors (n ≤ 10 with a combined score of ≥ 5). Circle diameter is an indicator of the confidence of the predictions.

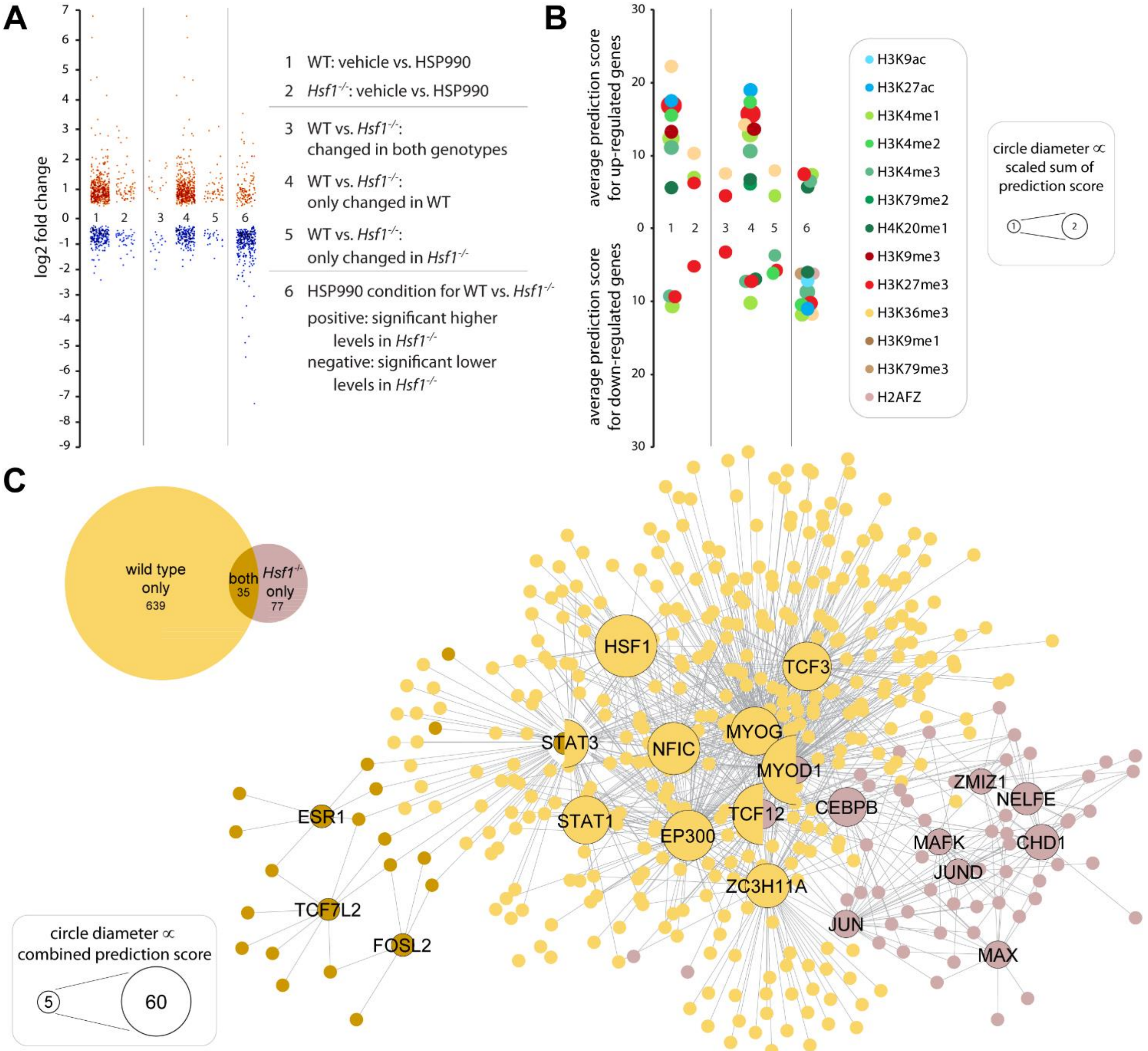

Figure S7. Differential systemic response to HSP90 inhibition in *Hsf1* knockout compared to wild type mice.

(A) Scatter plot showing the significant $\log_2$ fold transcriptome wide changes at 4 hours after HSP90 inhibition (HSP990) in quadriceps femoris muscle of *Hsf1* knockout (*Hsf1*$^{-/-}$) and wild type mice at 10-12 week of age. Lanes 1 and 2 represent the significantly regulated genes through heat shock in *Hsf1* knockout and wild type mice. Lanes 3 to 5 show the common (lane 3) and distinct (lanes 4 and 5) responses to treatment. Lane 6 compares HSP990 treated *Hsf1* knockout and wild type. Here, we corrected for differences due to the genotype by subtracting the $\log_2$ fold changes of significantly different genes (genotype) from their $\log_2$ induction value (HSP990). Only genes with a resulting fold change of ≥ 1.25 were considered for further analysis. (B) Chromatin mark predictions for genes shown in (A). Only significantly enriched chromatin marks ($p < 0.001$) were considered. We used the combined score, which is the product of the p-value with the z-score of the deviation from the expected rank, as a measure for prediction quality. Together, the average (y-axis) and the sum (circle diameter) of the combined scores are a good indicator of the confidence in the chromatin mark predictions. (C) Venn diagram and transcription factor network of common and distinct responses to HSP90 inhibition in *Hsf1* knockout and wild type mice. Data correspond to lanes 3, 4 and 5 in (A) and (B). To predict upstream regulators, we created gene lists for significantly regulated (up and down combined) genes for each condition and used the ENCODE transcription factor ChIP-seq database (2015) to identify the significantly enriched transcription factors (n ≤ 10 with a combined score of ≥ 5). RNA polymerase II hits were filtered out. Circle diameter is an indicator of the confidence of the predictions.

Supplementary Tables

Table S1. Gene ontology enrichment analysis for heat shock induction in wild type vs. R6/2 mice. Related to Table 1.

induction	regulation	genes	regulators (1: ChEA, 2: ENCODE)	pathways (1: WIKIPathways, 2: Reactome)	gene ontologies (biological process)	kinases	chromatin marks
both	up	77	1: CLOCK 51.62, HSF1 34.27, ESR1 32.55, EGR1 29.22, SUZ12 27.58<br>2: not significant	1: AGE/RAGE pathway 5.5<br>2: HSF1 activation 11.22, Attenuation phase (of HSF1) 11.13	cellular response to lipid 19.9, cellular response to hormone stimulus 13.97, positive regulation of reproductive process 7.67, response to temperature stimulus 7.51, negative regulation of transport 7.46	AKT1 19.37<br>RAF1 12.62<br>IRAK4 11.54<br>ROCK1 10.81<br>CDK8 9.51	H3K4me1 9.65<br>H3K9me3 8.15<br>H3K27me3 7.51
	down	10	1: EP300 19.44, TRIM28 16.47, VDR 12.16, GATA2 10.65, KLF4 9.13<br>2: not significant	1: TP53 Network 5.02<br>2: Signaling by Leptin 6.83, Death Receptor Signalling 5.73, Signaling by Retinoic Acid 5.55	negative regulation of cartilage development 11.66, response to leptin 6.85, cellular response to retinoic acid 6.53, response to acid chemical 6.52, negative regulation of protein phosphorylation 6.46	not significant	not significant
only WT	up	493	1: HSF1 265.41, CLOCK 262.11, ESR1 177.19, MYB 141.14, TCFAP2C 135,63<br>2: MYOD1 57.43, HSF1 47.24, MYOG 41.09, CHD1 33.71, EP300 33.09	1: Adipogenesis 23.32, Diurnally Regulated Genes with Circadian Orthologs 6.12, Aryl Hydrocarbon Receptor 5.49<br>2: Attenuation phase (of HSF1) 11.65, HSF1-dependent transactivation 9.89	response to topologically incorrect protein 33.65, regulation of p38MAPK cascade 16.97, regulation of vasculature development 13.21, positive regulation of protein kinase activity 11.84, muscle system process 11.22	IRAK4 109.88<br>ILK 78.68<br>IGF1R 46.44<br>EGFR 44.36<br>ITK 43.76	H3K4me1 27.36<br>H3K27me3 19.93<br>H3K4me3 19.27
	down	254	1: WT1 89.51, NANOG 78.48, POU5F1 71.03, ZFP281 65.03, MTF2 58.67<br>2: EP300 13.68, MAZ 13.46, MYOD1 11.2, MYOG 11.02, TCF3 9.87	1: Pluripotency 5.79<br>2: Signaling by BMP 14.42, Extracellular matrix organization 7.92, Oncogene Induced Senescence 7.75, Glycosaminoglycan metabolism 7.70, Integrin cell surface interactions 5.5	receptor protein serine/threonine kinase signaling pathway 18.53, BMP signaling pathway 18.05, cellular response to lipid 18.02, positive regulation of endothelial cell migration 17.79, regulation of smooth muscle cell migration 15.82	IRAK4 38.98<br>FGFR1 28.87<br>CDK8 18.53<br>BMPR2 10.65<br>TYK2 8.48	H3K27me3 32.03<br>H3K4me3 23.46<br>H3K4me1 15.92
only R6/2	up	13	1: STAT3 26.27, CLOCK 10.63, EGR1 10.07, SUZ12 8.61, CDX2 8.02<br>2: not significant	1: not significant<br>2: not significant	nitric oxide mediated signal transduction 10.37, small GTPase mediated signal transduction 8.79, regulation of Ras GTPase activity 6.1	IRAK4 12.57<br>BMPR1A 7.41	not significant
	down	9	1: not significant<br>2: not significant	1: Striated Muscle Contraction 17.02<br>2: Striated Muscle Contraction 21.6	actin-myosin filament sliding 16.65	FGFR1 5.80	not significant

Upstream regulators were predicted using the ChIP-x Enrichment Analysis (1: ChEA) and ENCODE transcription factor ChIP-seq database 2015 (2: ENCODE). Chromatin marks were predicted using the ENCODE histone modifications database 2015. Only the top non-redundant significantly enriched terms followed by their combined score are shown.

Table S2. Gene ontology enrichment for heat shock treatment in wild type vs. R6/2 mice. Related to Table 1.

induction	genes	regulators (1: ChEA, 2: ENCODE)	pathways (1: WIKIPathways, 2: Reactome)	gene ontologies (biological process)	kinases	chromatin marks
higher in WT	297	1: HSF1 192.57, CLOCK 73.44, TRIM28 61.1, WT1 55.45, TCF21 55.23<br>2: TCF12 48.90, MYOD1 34.38, HSF1 28.06, MYOG 16.86, MYOD1 12.56	1: Striated Muscle Contraction 20.96, Biogenic Amine Synthesis 7.05, Hypertrophy Model 5.58<br>2: Striated Muscle Contraction 16.74, HSF1 activation 15.85, Attenuation phase (of HSF1) 15.73, Metabolism of amino acids and derivatives 7.63, Translocation of GLUT4 to the plasma membrane 6.03	response to topologically incorrect protein 29.24, muscle filament sliding 28.04, response to hexose 7.46, regulation of ATP catabolic process 7.11, glucan metabolic process 7.09	ILK 87.62<br>AKT1 29.33<br>HUNK 28.7<br>CDK8 23.75<br>JAK2 23.22	H3K27me3 19.1<br>H3K4me1 16.8<br>H3K4me2 9.1
higher in R6/2	197	1: WT1 74.38, PPARG 56.55, SUZ12 56.37, SALL4 50.93, MTF2 48.94<br>2: UBTF 8.0, EZH2 7.54, TCF3 6.97, MYOD1 6.8, RXRA 6.64	1: not significant<br>2: not significant	not significant	BMPR1A 18.25<br>IGF1R 12.73<br>BMPR2 11.57<br>CDK8 10.66<br>FGFR1 8.05	H3K27me3 15.33<br>H3K4me1 12.96<br>H3K9me3 12.94

Upstream regulators were predicted using the ChIP-x Enrichment Analysis (1: ChEA) and ENCODE transcription factor ChIP-seq database 2015 (2: ENCODE). Chromatin marks were predicted using the ENCODE histone modifications database 2015. Only the top non-redundant significantly enriched terms followed by their combined score are shown.

Table S3. Gene ontology enrichment analysis for HSP990 induction in wild type vs. R6/2 mice. Related to Table 2.

induction	regulation	genes	regulators (1: ChEA, 2: ENCODE)	pathways (1: WIKIPathways, 2: Reactome)	gene ontologies (biological process)	kinases	chromatin marks
both	up	82	1: HSF1 287.64, CLOCK 44.03, E2F1 37.77, MYC 33.51, TRIM28 26.46<br>2: HSF1 76.6, EP300 14.6, CTCF 14.19, STAT1 13.22, ARID3A 12.85	1: MAPK Signaling Pathway 5.63, Apoptosis Modulation and Signaling 5.23, IL-6 signaling Pathway 5.11<br>2: HSF1 activation 52.25, Attenuation phase (of HSF1) 52.13	protein folding 50.25, regulation of apoptotic signaling pathway 10.56, immune response-activating signal transduction 9.07, negative regulation of phosphorylation 8.17, positive regulation of ATP catabolic process 8.1	CDK8 27.16<br>ILK 26.41<br>SNRK 16.12<br>ATM 15.76<br>IRAK4 12.38	H3K36me3 7.42<br>H3K79me2 6.86<br>H3K27ac 6.39
	down	81	1: TAF7L 50.04, CLOCK 49.36, NANOG 41.85, FOXP2 34.17, CDX2 33.88<br>2: CTCF 8.5	1: not significant<br>2: not significant	not significant	HUNK 9.59<br>KSR2 9.26<br>ROCK2 8.82<br>IGF1R 7.17<br>KSR1 6.24	H3K4me1 11.46<br>H3K9me3 7.85<br>H3K27me3 7.74
only WT	up	397	1: ESR1 185.93, HSF1 155.01, SUZ12 125.28, EP300 124.68, TCF21 121.95<br>2: TCF12 56.02, MYOD1 47.18, GATA3 35.0, ZC3H11A 31.22, NFIC 30.68	1: Focal Adhesion 31.24, Inflammatory Response Pathway 20.03, protein-protein interactions in the podocyte 7.61, Type II interferon signaling (IFNG) 7.59, Senescence and Autophagy 6.48<br>2: Extracellular matrix organization 66.99, Collagen biosynthesis and modifying enzymes 29.33, Non-integrin membrane-ECM interactions 13.99, Integrin cell surface interactions 10.74, ECM proteoglycans 9.93	extracellular matrix organization 88.47, collagen fibril organization 36.53, response to wounding 21.87, response to acid chemical 20.16, endodermal cell differentiation 17.36	IRAK4 143.3<br>KSR1 85.83<br>BMPR2 55.78<br>CDK8 46.19<br>FGFR1 39.56	H3K27me3 41.29<br>H3K27ac 28.14<br>H3K4me1 27.13
	down	114	1: CLOCK 41.54, TAF7L 37.67, EP300 35.35, KLF4 35.23, TCFAP2C 32.8<br>2: RFX5 9.75, TCF7L2 7.71, TFAP2A 7.61, RFX5 6.21, MAFK 5.49	1: not significant<br>2: Smooth Muscle Contraction 5.84, Fatty acid, triacylglycerol, and ketone body metabolism 5.27	organic acid transport 8.32, muscle contraction 6.78	ILK 19.86<br>IRAK4 17.43<br>IGF1R 8.28<br>PDK1 7.48<br>AKT1 5.88	H3K4me1 5.76
only R6/2	up	66	1: EZH2 25.79, RNF2 25.65, PPARG 21.62, PHC1 21.45, MTF2 20.08<br>2: not significant	1: Adipogenesis 7.97<br>2: not significant	response to peptide 9.14, negative regulation of tumor necrosis factor production 8.37, regulation of endothelial cell migration 6.98, carbohydrate derivative transport 6.5, nucleoside transmembrane transport 6.35	CDK8 6.41<br>ROCK2 6.33<br>FGFR1 5.9	H3K27me3 7.15<br>H3K4me1 6.89<br>H3K4me3 5.51
	down	169	1: ESR1 61.57, MTF2 52.28, ZNF217 48.95, CLOCK 45.63, NANOG 43.85<br>2: NFIC 14.24, RAD21 10.99, TCF12 10.83, EP300 10.44, CTCF 10.1	1: EGF/EGFR Signaling Pathway 5.82, Adipogenesis 5.71, Signaling Pathways in Glioblastoma 5.55<br>2: not significant	regulation of response to wounding 19.29, regulation of inflammatory response 18.83, regulation of vasculature development 11.39, response to oxidative stress 10.52, positive regulation of phospholipase C activity 9.99	IGF1R 28.05<br>IRAK4 23.71<br>KSR2 21.46<br>HUNK 11.2<br>ALK 10.3	H3K27me3 35.86<br>H3K4me1 23.8<br>H3K9me3 8.79

Upstream regulators were predicted using the ChIP-x Enrichment Analysis (1: ChEA) and ENCODE transcription factor ChIP-seq database 2015 (2: ENCODE). Chromatin marks were predicted using the ENCODE histone modifications database 2015. Only the top non-redundant significantly enriched terms followed by their combined score are shown.

Table S4. Gene ontology enrichment for HSP990 treatment in wild type vs. R6/2 mice. Related to Table 2.

induction	genes	regulators (1: ChEA, 2: ENCODE)	pathways (1: WIKIPathways, 2: Reactome)	gene ontologies (biological process)	kinases	chromatin marks
higher in WT	387	1: EP300 110.34, HSF1 104.32, MTF2 91.0, CLOCK 90.33, TCF21 88.45<br>2: TCF12 39.81, MYOD1 37.01, FOSL2 31.87, TCF3 27.86, NFIC 27.49	1: Focal Adhesion 18.61, Spinal Cord Injury 16.74, Diurnally Regulated Genes with Circadian Orthologs 7.6, Inflammatory Response Pathway 5.1<br>2: Extracellular matrix organization 57.15, Collagen formation 35.32, Attenuation phase (of HSF1) 11.88, O-glycosylation of TSR domain-containing proteins 11.65, HSF1 activation 11.39	extracellular matrix organization 63.41, collagen metabolic process 36.41, protein refolding 24.51, angiogenesis 14.25, response to wounding 8.3	IRAK4 87.93<br>CDK8 55.52<br>KSR1 41.97<br>BMPR1A 30.81<br>FGFR1 29.93	H3K27me3 47.47<br>H3K4me1 30.59<br>H3K9me3 27.39
higher in R6/2	455	1: ESRRB 164.39, POU5F1 118.35, E2F1 100.62, NANOG 100.61, TRIM28 93.54<br>2: MYOD1 51.69, MYOG 50.61, SRF 46.77, TCF12 33.81, UBTF 29.18	1: Striated Muscle Contraction 27.04, Electron Transport Chain 20.5, TCA Cycle 13.43, Glycolysis and Gluconeogenesis 12.17, Oxidative phosphorylation 8.64<br>2: The citric acid (TCA) cycle and respiratory electron transport 37.2, Striated Muscle Contraction 23.2, Glucose metabolism 11.46	muscle filament sliding 41.56, generation of precursor metabolites and energy 40.37, glycolytic process 13.19, pattern specification process 10.22, mitochondrion organization 10.2	ILK 92.21<br>BMPR2 57.05<br>AKT2 40.63<br>IRAK4 23.79<br>ERBB2 17.69	H3K4me3 34.96<br>H3K27ac 32.7<br>H3K36me3 20.5

Upstream regulators were predicted using the ChIP-x Enrichment Analysis (1: ChEA) and ENCODE transcription factor ChIP-seq database 2015 (2: ENCODE). Chromatin marks were predicted using the ENCODE histone modifications database 2015. Only the top non-redundant significantly enriched terms followed by their combined score are shown.

Table S5. Gene ontology enrichment analysis for heat shock vs. HSP990 treatment in wild type mice. Related to Tables 1 and 2.

induction	genes	regulators (1: ChEA, 2: ENCODE)	pathways (1: WIKIPathways, 2: Reactome)	gene ontologies (biological process)	kinases	chromatin marks
higher levels with HSP990[1]	747	1: EP300 241.61, HSF1 233.85, ESR1 229.74, MTF2 173.07, TCF3 167.26<br>2: MYOD1 73.1, TCF3 65.38, GATA2 59.98, TCF12 59.97, MYOG 47.86	1: Focal Adhesion 14.88, protein-protein interactions in the podocyte 8.71, Id Signaling Pathway 7.39, Endochondral Ossification 7.25, Integrated Pancreatic Cancer Pathway 6.38<br>2: Extracellular matrix organization 22.1, Assembly of collagen fibrils and other multimeric structures 14.3, Signaling by PDGF 13.74, PI3K events in ERBB2/4 & AKT signaling 11.2, Signaling by SCF-KIT 10.66	angiogenesis 40.04, positive regulation of cell migration 37.48, regulation of protein serine/threonine kinase activity 36.16, regulation of extrinsic apoptotic signaling pathway 24.0, response to wounding 22.95	IRAK4 217.81<br>CDK8 134.72<br>KSR1 77.8<br>BMPR2 61.47<br>IGF1R 47.82	H3K27me3 53.44<br>H3K27ac 47.58<br>H3K4me3 34.46
higher levels with heat shock[2]	719	1: CLOCK 197.76, TCFAP2C 162.7, MYC 160.66, CCND1 146.39, E2F1 141.54<br>2: TCF12 69.97, MYOD1 65.47, MYOG 61.28, CTCF 57.57, EP300 51.85	1: Electron Transport Chain 10.69<br>2: The citric acid (TCA) cycle and respiratory electron transport 20.58	generation of precursor metabolites and energy 15.23, response to oxidative stress 15.22, muscle system process 14.81, negative regulation of intracellular signal transduction 13.02, regulation of p38MAPK cascade 12.61	ILK 145.99<br>IRAK4 92.22<br>KSR2 52.15<br>IGF1R 43.96<br>HUNK 40.68	H3K36me3 50.94<br>H3K4me3 32.97<br>H3K79me2 32.71

Upstream regulators were predicted using the ChIP-x Enrichment Analysis (1: ChEA) and ENCODE transcription factor ChIP-seq database 2015 (2: ENCODE). Chromatin marks were predicted using the ENCODE histone modifications database 2015. Only the top non-redundant significantly enriched terms followed by their combined score are shown. [1]Can be higher induced by HSP990 than by heat shock, or not changed by HSP990, but repressed through heat shock. [2]Can be higher induced by heat shock than by HSP990, or not changed by heat shock, but repressed through HSP990.

Table S6. Gene ontology enrichment analysis for heat shock induction in wild type vs. *Hsf1* knockout mice. Related to Table 1.

induction	regulation	genes	regulators (1: ChEA, 2: ENCODE)	pathways (1: WIKIPathways, 2: Reactome)	gene ontologies (biological process)	kinases	chromatin marks
both	up	168	1: ESR1 132.01, CLOCK 97.56, PPARG 67.87, ELK3 63.63, MTF2 63.1<br>2: ESR1 18.24, TFAP2A 17.82, MYOD1 17.69, NR3C1 17.47, NFIC 17.12	1: Adipogenesis 15.69, Oncostatin M Signaling Pathway 10.32<br>2: not significant	regulation of p38MAPK cascade 9.11, retina vasculature morphogenesis in camera-type eye 8.92, cellular response to lipid 8.71, cellular response to reactive nitrogen species 8.64, intrinsic apoptotic signaling pathway in response to DNA damage by p53 class mediator 8.53	IRAK4 49.45<br>HUNK 25.21<br>KSR1 20.12<br>IGF1R 17.09<br>EGFR 12.9	H3K4me1 20.67<br>H3K27me3 13.24<br>H3K9me3 10.53
	down	37	1: OLIG2 26.49, SMARCA4 20.94, TP53 19.16, GABPA 18.5, NFE2L2 18.4<br>2: not significant	1: Angiogenesis 6.55<br>2: not significant	negative regulation of endothelial cell apoptotic process 8.15, positive regulation of focal adhesion assembly 7.85, regulation of vascular permeability 7.38, hippo signaling 7.11, regulation of endothelial cell proliferation 6.67	CDK8 10.89<br>IRAK4 10.37<br>FGFR1 7.49<br>ROCK2 5.32	H3K27me3 15.46<br>H3K9me3 13.94<br>H3K4me3 7.81
only WT	up	402	1: HSF1 249.85, CLOCK 218.61, TCFAP2C 105.45, NANOG 98.34, MYB 96.59<br>2: HSF1 54.05, MYOG 36.47, MYOD1 33.95, CHD1 26.87, ZMIZ1 24.39	1: Adipogenesis 6.62, Diurnally Regulated Genes with Circadian Orthologs 5.12<br>2: Attenuation phase (of HSF1) 34.4, HSF1 activation 29.43	response to topologically incorrect protein 46.33, muscle system process 13.67, regulation of protein ubiquitination 10.9, positive regulation of kinase activity 10.81, glucan biosynthetic process 10.71	ILK 84.08<br>IRAK4 74.05<br>ITK 42.77<br>IGF1R 36.48<br>EGFR 29.71	H3K4me1 22.59<br>H3K27me3 15.77<br>H3K4me3 15.27
	down	227	1: WT1 71.41, POU5F1 65.32, NANOG 64.29, ZFP281 61.45, EP300 58.46<br>2: MAZ 14.92, MYOD1 9.03, UBTF 8.74, MYOG 8.55, TCF3 8.25	1: Pluripotency 7.67<br>2: Signaling by BMP 15.98, Non-integrin membrane-ECM interactions 9.25, Diseases associated with glycosaminoglycan metabolism 9.25, Extracellular matrix organization 7.28, Chondroitin sulfate/dermatan sulfate metabolism 5.22	cellular response to lipid 27.09, receptor protein serine/threonine kinase signaling pathway 23.27, BMP signaling pathway 21.07, regulation of smooth muscle cell migration 16.91, regulation of peptidyl-tyrosine phosphorylation 13.35	IRAK4 29.62<br>FGFR1 22.29<br>CDK8 11.19<br>BMPR2 7.19<br>KSR2 7.02	H3K27me3 22.71<br>H3K4me1 17.34<br>H3K4me3 16.13
only $Hsf1^{-/-}$	up	70	1: STAT3 46.33, NANOG 40.11, YAP1 27.15, NR1I2 25.34, RCOR2 24.96<br>2: MYOD1 5.61	1: Diurnally Regulated Genes with Circadian Orthologs 5.09<br>2: not significant	regulation of blood circulation 16.74, muscle system process 9.31, negative regulation of cellular carbohydrate metabolic process 7.27, response to oxygen levels 7.27, negative regulation of transport 7.2	IRAK4 19.58<br>KSR1 14.18<br>CDK8 13.13<br>BTK 9.94<br>KSR2 6.88	H3K9me3 9.21<br>H3K27me3 8.68<br>H3K4me1 6.15
	down	67	1: TCF3 28.9, CLOCK 27.73, PPARG 21.31, HOXB4 20.4, ZFP281 20.17<br>2: HDAC6 7.93, REST 6.36	1: Striated Muscle Contraction 16.18, SIDS Susceptibility Pathways 8.32, Adipogenesis genes 6.93, PPAR signaling pathway 6.92<br>2: Striated Muscle Contraction 18.68	actin-myosin filament sliding 21.03, fat cell differentiation 12.2, positive regulation of osteoclast differentiation 12.02, lipid storage 11.54, muscle tissue morphogenesis 11.12	ILK 16.67<br>FGFR1 11.08<br>CDK8 10.77<br>HUNK 8.72<br>AKT2 8.46	H3K27me3 5.22

Upstream regulators were predicted using the ChIP-x Enrichment Analysis (1: ChEA) and ENCODE transcription factor ChIP-seq database 2015 (2: ENCODE). Chromatin marks were predicted using the ENCODE histone modifications database 2015. Only the top non-redundant significantly enriched terms followed by their combined score are shown.

Table S7. Gene ontology enrichment analysis for HSP990 treatment in wild type vs. *Hsf1* knockout mice. Related to Table 2.

induction	regulation	genes	regulators (1: ChEA, 2: ENCODE)	pathways (1: WIKIPathways, 2: Reactome)	gene ontologies (biological process)	kinases	chromatin marks
both	up	18	1: STAT3 36.5, GATA4 31.91, ZNF217 21.27, SALL4 16.88, SOX2 14.95<br>2: FOSL2 12.12, FOS 10.82, TEAD4 10.62, STAT3 7.55, FOSL1 7.49	1: miR-targeted genes in leukocytes 10.8, Hypertrophy Model 8.74, miR-targeted genes in epithelium 7.73<br>2: Amino acid transport across the plasma membrane 15.92, O-glycosylation of TSR domain-containing proteins 8.27	amino acid transmembrane transport 10.55, anion transmembrane transport 10.31, cellular protein catabolic process 8.43, regulation of granulocyte chemotaxis 7.53, regulation of extrinsic apoptotic signaling pathway 6.86	IRAK4 9.8<br>KSR1 9.75<br>ALK 5.48<br>ERBB2 5.32	H3K36me3 7.57
	down	17	1: CLOCK 14.17, HOXA2 10.71, SCL 9.13, SMARCA4 8.95, MYB 8.75<br>2: not significant	1: MAPK Signaling Pathway 7.34<br>2: not significant	regulation of endothelial cell differentiation 10.49, Rho protein signal transduction 5.82, embryo development 5.42, positive regulation of MAPK cascade 5.4, negative regulation of intracellular signal transduction 5.36	KSR2 5.75	not significant
only WT	up	461	1: HSF1 386.77, ESR1 174.05, CLOCK 143.01, EP300 129.11, TCF21 117.95<br>2: HSF1 69.18, TCF12 57.22, MYOD1 48.83, GATA3 36.44, EP300 35.31	1: Focal Adhesion 25.85, Inflammatory Response Pathway 13.74, IL-2 Signaling Pathway 10.43, Type II interferon signaling (IFNG) 9.25, Adipogenesis genes 8.56<br>2: Extracellular matrix organization 58.54, Collagen biosynthesis and modifying enzymes 33.77, Attenuation phase (of HSF1) 26.16, HSF1 activation 25.2, Interferon alpha/beta signaling 11.86	extracellular matrix organization 96.82, protein folding 45.63, collagen fibril organization 44.84, response to wounding 24.37, positive regulation of cell migration 20.41	IRAK4 147.75<br>KSR1 75.53<br>CDK8 67.29<br>BMPR2 59.08<br>FGFR1 43.63	H3K27me3 32.85<br>H3K27ac 30.24<br>H3K4me1 24.32
	down	178	1: TAF7L 85.28, CLOCK 75.34, FOXP2 62.77, MEF2A 52.26, VDR 45.55<br>2: RFX5 19.38, E2F1 12.77, EP300 12.7, MAZ 10.18, TCF7L2 9.44	1: not significant<br>2: not significant	organic acid transport 7.23, negative regulation of lipid storage 5.25	ILK 24.23<br>IRAK4 21.9<br>HUNK 16.03<br>IGF1R 13.24<br>AKT1 10.12	H3K4me1 12.08<br>H3K27me3 8.85<br>H3K4me3 8.24
only *Hsf1*$^{-/-}$	up	53	1: ATF3 32.04, RARG 30.83, TAF7L 30.45, NANOG 27.14, GATA1 26.11<br>2: CEBPB 36.7, JUN 21.25, CHD1 20.46, NELFE 19.24, MAX 16.01	1: MAPK Signaling Pathway 9.18<br>2: PERK regulates gene expression 17.0, ATF4 activates genes 16.82, Amino acid synthesis and interconversion 11.87, Unfolded Protein Response (UPR) 9.13, ATF6-alpha activates chaperones 5.92	cellular response to topologically incorrect protein 19.05, response to endoplasmic reticulum stress 15.68, ER-nucleus signaling pathway 14.37, cellular amino acid metabolic process 13.21, apoptotic signaling pathway 12.64	EGFR 30.23<br>KSR1 27.55<br>ALK 18.17<br>SNRK 15.58<br>BMPR1A 15.01	H3K36me3 7.97
	down	24	1: SMAD3 16.91, KLF2/4/5 14.3, SMAD4 13.85, NOTCH1 13.76, ZFP281 13.44<br>2: not significant	1: Striated Muscle Contraction 12.44<br>2: Striated Muscle Contraction 10.58, Basigin interactions 5.29	negative regulation of ERK1 and ERK2 cascade 13.81, muscle filament sliding 13.17, regulation of response to wounding 11.91, negative regulation of MAPK cascade 7.04, regulation of cell adhesion 6.92	not significant	H3K4me2 6.17<br>H3K27me3 5.75

Upstream regulators were predicted using the ChIP-x Enrichment Analysis (1: ChEA) and ENCODE transcription factor ChIP-seq database 2015 (2: ENCODE). Chromatin marks were predicted using the ENCODE histone modifications database 2015. Only the top non-redundant significantly enriched terms followed by their combined score are shown.

Table S8. Gene ontology enrichment for heat shock treatment in wild type vs. *Hsf1* knockout mice. Related to Table 1.

induction	genes	regulators (1: ChEA, 2: ENCODE)	pathways (1: WIKIPathways, 2: Reactome)	gene ontologies (biological process)	kinases	chromatin marks
higher in WT	249	1: HSF1 356.43, CLOCK 100.92, E2F1 89.96, MYC 81.35, TAF7L 66.29<br>2: HSF1 98.17, IRF1 25.14, EP300 24.27, PBX3 23.99, NFYB 20.61	1: Diurnally Regulated Genes with Circadian Orthologs 5.87, Glycolysis and Gluconeogenesis 5.67<br>2: Attenuation phase (of HSF1) 40.04, HSF1 activation 39.81, CCT/TriC 8.78	protein folding 64.3, muscle system process 23.35, cell recognition 9.14, adipose tissue development 6.33, positive regulation of nitric oxide biosynthetic process 6.26	ILK 109.75<br>ATM 43.19<br>ERBB2 24.41<br>BMPR2 24.65<br>IRAK4 22.44	H3K4me3 18.44<br>H4K20me1 17.97<br>H3K4me1 17.01
higher in *Hsf1*$^{-/-}$	155	1: ESR1 58.73, ZNF217 56.97, NANOG 44.79, SOX2 44.3, SMARCA4 43.58<br>2: UBTF 22.07, SMARCC2 9.19, CTCF 8.79, MYOD1 7.84, SMARCC1 7.76	1: not significant<br>2: SHC-related events triggered by IGF1R 7.78, Signaling by BMP 6.77, Axon guidance 6.34, Gap junction degradation 6.01, Adenylate cyclase activating pathway 5.76	regulation of ossification 10.8, neuron projection guidance 9.16, positive regulation of cell migration 8.61, negative regulation of stem cell proliferation 8.44, astrocyte development 8.24	IRAK4 29.83<br>CDK8 18.58<br>KSR1 11.21<br>BMPR2 11.2<br>ERBB3 11.11	H3K9me3 12.23<br>H3K27me3 10.96<br>H3K79me2 10.78

Upstream regulators were predicted using the ChIP-x Enrichment Analysis (1: ChEA) and ENCODE transcription factor ChIP-seq database 2015 (2: ENCODE). Chromatin marks were predicted using the ENCODE histone modifications database 2015. Only the top non-redundant significantly enriched terms followed by their combined score are shown.

Table S9. Gene ontology enrichment for HSP990 treatment in wild type vs. *Hsf1* knockout mice. Related to Table 2.

induction	genes	regulators (1: ChEA, 2: ENCODE)	pathways (1: WIKIPathways, 2: Reactome)	gene ontologies (biological process)	kinases	chromatin marks
higher in WT	286	1: HSF1 499.23, CLOCK 79.38, TRIM28 71.51, ESR1 68.48, E2F1 66.79<br>2: HSF1 90.15, MYOG 33.96, MYOD1 27.23, TCF3 25.68, EP300 24.46	1: not significant<br>2: Attenuation phase (of HSF1) 36.59, HSF1 activation 36.35, CCT/TriC 12.12, Collagen formation 8.6, Extracellular matrix organization 8.25	protein folding 69.68, regulation of cytokine production 14.01, extracellular matrix organization 13.77, muscle system process 9.81, negative regulation of TGFbeta receptor signaling pathway 8.21	ILK 73.08<br>IRAK4 48.54<br>BMPR2 29.3<br>EGFR 28.16<br>CDK8 25.74	H3K4me1 20.23<br>H3K36me3 19.98<br>H3K27me3 16.21
higher in *Hsf1*$^{-/-}$	121	1: NR1H3 50.36, NANOG 38.1, TAF7L 34.7, MYC 33.99, CLOCK 33.92<br>2: CEBPB 62.83, MAX 26.26, EP300 17.54, TFAP2A 17.24, NR3C1 15.0	1: Aryl Hydrocarbon Receptor 5.64, TP53 Network 5.33<br>2: Cytosolic tRNA aminoacylation 16.14, Amino acid synthesis and interconversion 12.78, Signaling by the B Cell Receptor (BCR) 7.29, Amino acid and oligopeptide SLC transporters 6.65	cellular amino acid metabolic process 10.75, tRNA aminoacylation for protein translation 10.17, regulation of translational fidelity 9.43, regulation of stress-activated protein kinase signaling cascade 8.43, response to acid chemical 7.58	EGFR 32.31<br>IRAK4 21.13<br>JAK2 17.78<br>CDK8 12.86<br>ALK 11.51	H3K27me3 11.12<br>H3K4me1 8.77<br>H3K4me3 7.76

Upstream regulators were predicted using the ChIP-x Enrichment Analysis (1: ChEA) and ENCODE transcription factor ChIP-seq database 2015 (2: ENCODE). Chromatin marks were predicted using the ENCODE histone modifications database 2015. Only the top non-redundant significantly enriched terms followed by their combined score are shown.

Supplementary Materials and Methods

Table S10. Primers and Probes sequences

The sequences of primers and probes used in this study were as follows:

gene	forward primer	reverse primer	probe (5'-FAM, 3'-TAMRA)	supplier
Hspa1a/b	GGTGGTGCAGTCCGACATG	TTGGGCTTGTCGCCGT	CACTGGCCCTTCCAGGTGGTGAA	Eurofins
Dnajb1	CCCCATGCCATGTTTGCT	GCGCTGCCCAAAAAAGG	TCTTCGGTGGCAGAAACCCCTTTGA	Eurofins
Hspb1	CACTGGCAAGCACGAAGAAAG	GCGTGTATTTCCGGGTGAAG	ACCGAGAGATGTAGCCATGTTCGTCCTG	Eurofins
Hsf1	CGAGTGGGAACAGCTTCCA	ACTTGGGCAGCACCTCCTT	TTTGACCAGGGCCAGTT	Eurofins
Hsp90aa1	GGGCCCGCCTCTATATAAGG	GACCTCCTCCTCCTCCATTG		PrimerDesign[1]
Hsp90ab1	ACCTCAAAGAAGACCAGACAGA	GGATAGCCTATGAACTGCGAAT		PrimerDesign[1]
Hsph1	AACACAGCCCCAGGTACAA	TTTGCTTTCTTCTGAGGTAAGTTC		PrimerDesign[1]
Hspd1	GTCCCTGCTCTTGAAATTGCTA	GACTGCCACAACCTGAAGAC		PrimerDesign[1]
Hspe1	CAACGGTCGTGGCTGTGG	AGAACTTTATCTCCAACTTTCACACT		PrimerDesign[1]
Cryab	CCTCTTCTCAACAGCCACTTC	GTCCTTCTCCAAACGCATCTC		PrimerDesign[1]
Tcp1	TTGGAAGGCGAAGAAACTTTTGA	GCTCATCATCACAAATTCTCTCCT		PrimerDesign[1]
Hif1a	GATGTAATGTTTCCCTCTTCTAATGA	GCAGGATCAGCACTACTTCG		PrimerDesign[1]
Atp5b				PrimerDesign[2]
Sdha				PrimerDesign[2]
Actb				PrimerDesign[2]
Canx				PrimerDesign[2]
Ywhaz				PrimerDesign[2]
Ubc				PrimerDesign[2]
Gapdh				PrimerDesign[2]

[1]Probe sequences are not disclosed by PrimerDesign. [2]No information is disclosed for housekeeping genes by PrimerDesign.

Table S11. Antibodies information and dilutions

Information about antibodies and dilutions used in this study can be found in the following table:

antibody	catalogue number	supplier	dilution
HSP90	ADI-SPA-835	Stressgen	quad. fem: 1:1000; tib. ant.: 1:1000; cortex: 1:3000; liver 1:1000
HSP70	ADI-SPA-810	Stressgen	quad. fem: 1:750; tib. ant.: 1:750; cortex: 1:1000; liver 1:1000
HSP40	ADI-SPA-400	Stressgen	quad. fem: 1:750; tib. ant.: 1:750; cortex: 1:1000; liver 1:1000
HSP25	ADI-SPA-801	Stressgen	quad. fem: 1:1000; tib. ant.: 1:1000; cortex: 1:1000; liver 1:1000
TUBA1a/b	T9026	Sigma	quad. fem: 1:10000; tib. ant.: 1:10000; cortex: 1:40000; liver 1:15000
ATP5B	ab14730	Abcam	1:10000
HSF1	Ab81279	Abcam	1:1000
anti-mouse 680nm	926-68070	Licor	1:7500
anti-mouse 800nm	926-32210	Licor	1:7500
anti-rabbit 680nm	926-68071	Licor	1:7500
anti-rabbit 800nm	926-32211	Licor	1:7500
anti-rat	926-32219	Licor	1:7500

quad. fem. = *quadriceps femoris*; tib. ant. = *tibialis anterior*

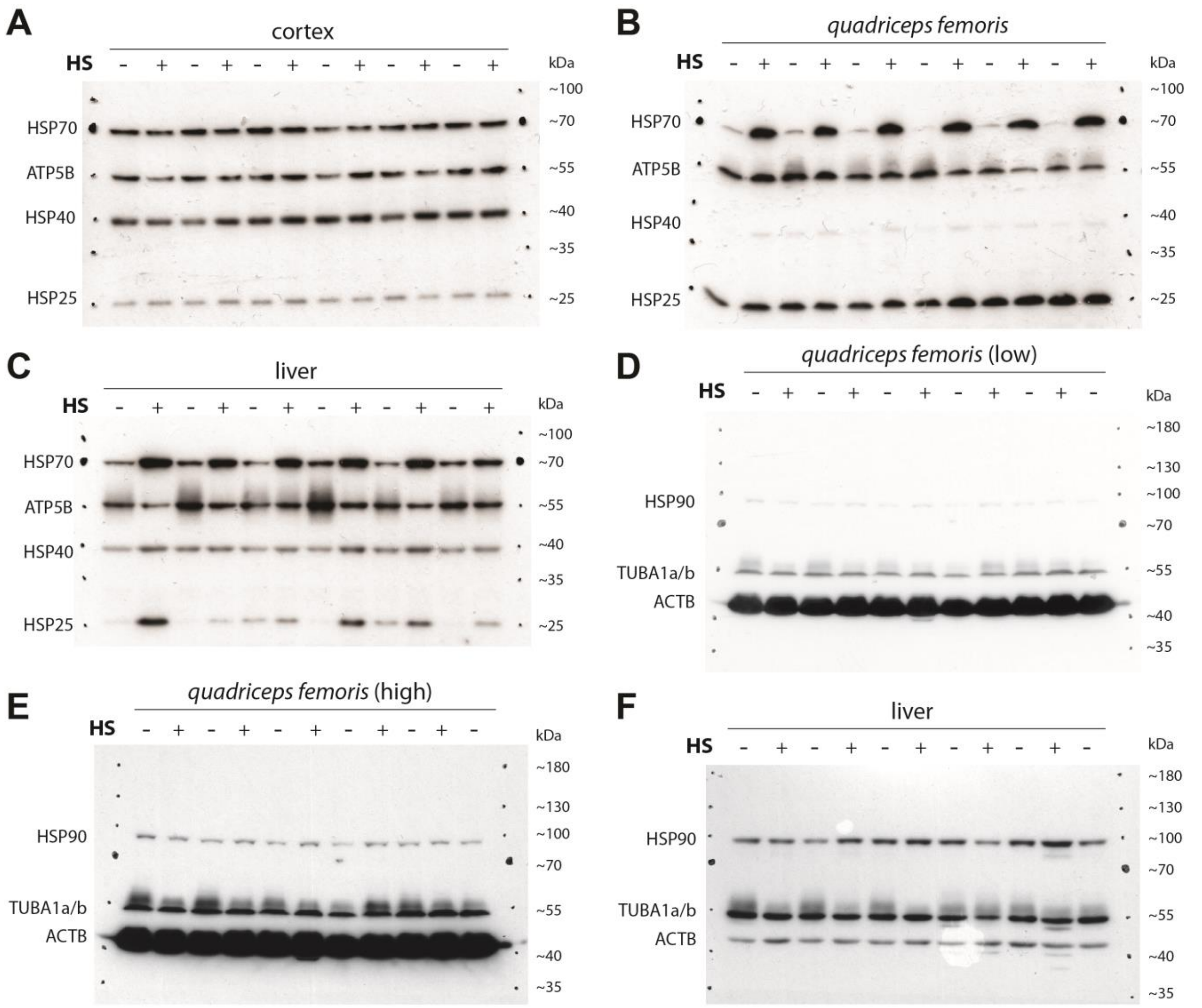

Figure S8. Antibody characterization

During the development of the *in vivo* heat shock (HS), we optimized antibody staining for the different tissues. Exemplary blots in wild type animals are shown for HSP70, HSP40 and HSP25 antibodies in (A) cortex, (B) *quadriceps femoris* and (C) liver and HSP90, tubulin (TUBA1a/b) and actin (ACTB) antibodies in (D) *quadriceps femoris* (low exposure), (E) *quadriceps femoris* (high exposure) and (F) liver. All antibodies are well characterized (see also Table S11), detect only a single band and exhibit very low background signals. For these reasons and to increase specificity even more, subsequently, blots were cut at the appropriate molecular weight for the respective protein of interest and the strips were incubated with individual antibodies.
